# Sustainable Synthesis of Dimethyl- and Diethyl Carbonate from CO_2_ in Batch and Continuous Flow—Lessons from Thermodynamics and the Importance of Catalyst Stability

**DOI:** 10.1021/acssuschemeng.2c00291

**Published:** 2022-04-12

**Authors:** Matthew F. O’Neill, Meenakshisundaram Sankar, Ulrich Hintermair

**Affiliations:** † Cardiff Catalysis Institute, School of Chemistry, Cardiff University, Cardiff CF10 3AT, United Kingdom; ‡ Centre for Sustainable and Circular Technologies, University of Bath, Bath BA2 7AY, United Kingdom

**Keywords:** direct synthesis of dialkyl carbonates, CO_2_ utilization, thermodynamics, continuous flow processing, supercritical fluids

## Abstract

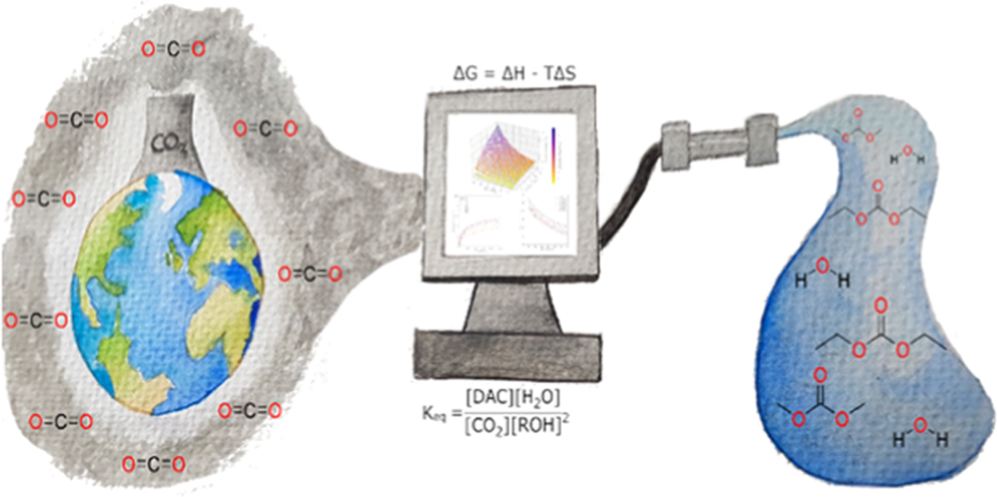

Equilibrium conversions for the direct condensation of MeOH and EtOH with CO_2_ to give dimethyl- and diethyl carbonate, respectively, have been calculated over a range of experimentally relevant conditions. The validity of these calculations has been verified in both batch and continuous flow experiments over a heterogeneous CeO_2_ catalyst. Operating under optimized conditions of 140 °C and 200 bar CO_2_, record productivities of 235 mmol/L·h DMC and 241 mmol/L·h DEC have been achieved using neat alcohol dissolved in a continuous flow of supercritical CO_2_. Using our thermodynamic model, we show that to achieve maximum product yield, both dialkyl carbonates and water should be continuously removed from the reactor instead of the conventionally used strategy of removing water alone, which is much less efficient. Catalyst stability rather than activity emerges as the prime limiting factor and should thus become the focus of future catalyst development.

## Abstract 2

Thermodynamic analyses and stability measurements are key to evaluating processes for sustainable CO_2_ utilization such as the catalytic formation of dialkyl carbonates.

## Introduction

**Scheme 1:**
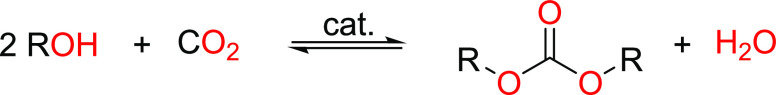
: General Reaction Scheme for the Catalytic Direct Synthesis of Linear Dialkyl Carbonates (DACs) from CO_2_

CO_2_ emissions are one of the biggest global issues affecting humanity due to their role in the warming of the earth’s atmosphere. A total of 37 billion tonnes were emitted globally in 2018, and the trend points upward.^1^ CO_2_ accounts for 65% of all greenhouse gas emissions,^2^ with transport and industry combined being responsible for 35%.^2^ Although unintentional, this vast production of CO_2_ is a consequence of our linear take-use-dispose exploitation of our planet’s fossil carbon reserves. More sustainable ways of fuel and chemical production and use are therefore pressing areas of research, and utilizing CO_2_ as a chemical feedstock is an integral part of realizing a more circular economy.^3^ Chemical valorization of CO_2_ is a key topic in this context,^4^ but is faced with a number of technological and fundamental (i.e., kinetic and thermodynamic) challenges.^5−8^ The redox-neutral incorporation of CO_2_ into organic carbonates (linear, cyclic, or polymeric) represents one of the most viable approaches to chemical CO_2_ utilization. Monomeric dialkyl carbonates (DACs) are useful compounds for a number of applications, from electrolyte solvents in Li-ion batteries, reagents in organic transformations, fuel additives, and green solvents.^9−11^ Dimethyl carbonate (DMC) is listed by the GSK solvent selection guide as a potential replacement for chlorinated solvents due to its low eco-toxicity, a property that diethyl carbonate (DEC) shares.^12^ The industrial production of DACs has historically used condensation of the corresponding alcohol with phosgene as a carbonyl source, although alternative methods such as urea alcolysis^13^ and oxidative carbonylation of alcohols^13,14^ are more attractive due to the avoidance of chloride waste and handling of hazardous phosgene. The oxidative carbonylation of alkenes has also been investigated for the production of cyclic carbonates.^15,16^ The utilization of CO_2_ in DAC synthesis via double condensation with alcohols (Scheme 1) is an appealing alternative,^17^ specifically for the production of DMC^18−21^ and DEC.^13,22−25^

A number of homogeneous and heterogeneous catalysts have been reported for this direct synthesis. Among the heterogeneous catalysts reported, CeO_2_ is by far the most common,^18,26−37^ with attempts to increase its effectiveness through surface modification.^38^ Mixed metal oxides such as Fe*_x_*Zr_1–*x*_O*_y_*,^39^ Ce*_x_*Al_1–*x*_O*_y_*,^28^ and even supported metal oxides such as Ce*_x_*Zr_1–*x*_O*_y_*/C^40^ have also been investigated, and homogeneous catalysts based on tin alkoxides^25,41^ have been tested with some success. The major difficulty with the direct DAC synthesis however is not the kinetics but the thermodynamics of the reaction. The redox-neutral double-condensation reaction is exothermic in the formation of the DAC, but overall the reaction is endergonic under typical reaction conditions (see below). Thus, for catalyst development, it has become customary to add dehydrating agents such as trimethoxy methane,^25,40^ 2-cyanopyridine,^30,37,42−45^ nitriles,^29,46,47^ epoxides,^48,49^ alkyl iodides,^50,51^ or carbodiimides^52^ to increase the driving force for DAC formation so that kinetic accelerations may be quantified more easily. Such conditions are less relevant to real-world application of course, as post-reaction regeneration of the dehydrating agent used would require at least the same amount of energy they add to the DAC synthesis, in addition to the difficulties of their separation, recovery, and reuse. It is also worth pointing out that all dehydrating agents used are synthetic products with significant carbon footprints from their own manufacturing, and their cost typically exceeds the value of the target DAC product by several orders of magnitude. More easily regenerable water removal approaches based on physical separation, such as membranes and molecular sieves, are thus more appealing from an applied perspective but, unfortunately, also much less effective in shifting the equilibrium position inside the reactor than chemical dehydration agents. Typically repeated reaction-removal cycles with long equilibration times are required^25^ due to the low quantities of water removed per cycle. Thus, while many stable^28^ and active^34^ catalysts have been reported for this important reaction under conditions rather irrelevant for application, the key process challenge of limiting equilibrium position under realistic conditions has received little attention so far.^24^ Here, we report thermodynamic equilibrium data for the free condensation of MeOH and EtOH with CO_2_ over a range of reaction conditions to provide a basis for comparing experimental results from catalytic reactions in batch and continuous flow. We demonstrate a productive continuous flow system with the ability to monitor catalyst stability, and propose a new process model based on repeated separation of both reaction products.

## Experimental Section

### General

All reagents were purchased from major commercial suppliers in the highest purity available. Methanol was distilled from magnesium turnings under inert atmosphere, and absolute ethanol was stored over molecular sieves under inert atmosphere. Methanol water content was determined to be 25 ppm by Karl Fischer titration (Mettler Toledo DL32). Commercial nanocrystalline cerium oxide (Alfa Aesar, 99.5%, 15–30 nm, BET surface area 30–50 m^2^/g) and industrial-grade CO_2_ (99.8%) were used as received.

### Analysis

NMR spectra were recorded in standard 5 mm glass tubes at room temperature on a 400 MHz instrument. Quantitative analysis of samples from catalytic experiments without dehydrating agents was performed on a Shimadzu GC-2010 plus equipped with a FID detector at 300 °C with a H_2_ flow rate of 40 mL/min, an air flow rate of 400 mL/min, and an argon makeup flow rate of 30 mL/min. Helium was used as a carrier gas at a constant linear velocity of 39.4 cm/s through a BP20 capillary column from SGE analytical science (30 m length, 0.25 mm ID, 0.5 μm film thickness). The sample (1 μL) was injected with a split ratio of 50 and an injector temperature of 250 °C. The column was held at 50 °C for 1 min, then the temperature ramped up to 70 °C at 3 °C/min, then to 180 °C at 7 °C/min, and finally up to 240 °C at 23 °C/min. For reactions with dehydrating agents, analysis was performed on a Varian 3900 GC equipped with a FID detector at 300 °C with a H_2_ flow rate of 40 mL/min, an air flow rate of 400 mL/min, and an argon makeup flow rate of 30 mL/min. Helium was used as a carrier gas at a constant linear velocity of 39.4 cm/s through a CP-Sil 5CB column from Agilent (50 m length, 0.32 mm ID, 5 μm film thickness). The sample (0.5 μL) was injected with a split ratio of 100 and an injector temperature of 300 °C. The column was held at 50 °C for 5 min, then the temperature ramped up to 70 °C at 3 °C/min, then to 180 °C at 7 °C/min, and finally up to 300 °C at 23 °C/min.

### Direct Batch Synthesis of DMC and DEC

Batch experiments were conducted in custom-made stainless steel autoclaves with a total volume of 20 mL. For reactions with dehydrating agents, 1 g of dry MeOH was added to a glass liner containing 0.03 g of CeO_2_, along with 0.5 mol equivalents of diisopropyl carbodiimide, 2-cyanopyridine, or trimethoxy methane and a Teflon-coated magnetic stirrer bar. The glass liner was inserted into a preheated autoclave, sealed, and pressurized with 50 bar CO_2_ at 40 °C. For reactions without dehydrating agents, 5 mL of dry alcohol was added to 0.3 g of nanocrystalline CeO_2_ in a glass liner containing a Teflon-coated magnetic stir bar. The glass liner was inserted into a preheated autoclave, which was then sealed and pressurized with 70 bar CO_2_ at 40 °C. The autoclaves were then placed into aluminum heating blocks set to the desired reaction temperature with stirring at 400 rpm. At the end of the reaction, the autoclaves were cooled to room temperature before being slowly depressurized. The liquid remaining in the glass liner was filtered, internal standard solutions were added, and the mixtures were analyzed by GC-FID. Yield is based on alcohol as the limiting reagent and is calculated using the following equation
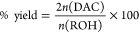
1where *n*(DAC) denotes the final moles of dialkyl carbonate and *n*(ROH) denotes the starting moles of alcohol.

### Continuous Flow Synthesis of DMC and DEC

A 10 cm piece of 1/2 inch stainless steel tubing was packed with 0.3 g of cerium oxide between two beds of glass wool. This was attached to a preheated continuous flow rig as described elsewhere.^53^ The reactor was wrapped in resistive heating tape (200 W, 120 cm) and temperature-controlled using a Eurotherm 2216 PID control box. The system was purged with 2 mL/min CO_2_ at 200 bar and 40 °C for 10 min. Then, the reactor containing the catalyst bed was heated up to the desired reaction temperature, and the reaction was started by the addition of liquid alcohol from an inert reservoir at room temperature pumped at 0.2 mL/min. A back-pressure regulator (JASCO BP-2080 Plus) was connected to an autosampler (GE Frac920). The degassed liquid (6 mL) eliminated by the back-pressure regulator (see Figure S6) was collected in 30 min intervals for 0.2 mL/min. Aliquots (1 mL) of these samples were taken, 0.12 mmol mesitylene was added to each, and the mixtures were analyzed by GC-FID. Productivity was calculated via eq 1, where *p* is the productivity, *n*(DAC) is the number of moles of dialkyl carbonate formed, *v* is the volume of the catalyst bed, and *t* is the reaction time.
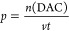
2

### DMC Hydrolysis

Hydrolysis experiments of DMC were performed using a stock solution consisting of 1 mL of DMC, 0.236 mL of H_2_O (adjusted to pH 3 with 1 M HNO_3_), 1 mL of DMSO (to homogenize the mixture), and 0.05 mL of d_6_-DMSO. Sealed J-Young’s NMR tubes were then loaded with 1 mL of the homogeneous solution and placed into an aluminum heating block set to the desired hydrolysis temperature. The mixture was periodically analyzed by quantitative ^1^H NMR (zg30, D1 = 1 s, NS = 16), and the MeOH peak area at 3.18 ppm was monitored to calculate DMC conversion.

### Thermodynamic Calculations

Values for Δ*H*_f_^0^ and *S*^0^ for DEC and DMC were obtained from the literature (see Table S3).^13,23^

Δ*H*_r_^0^ and Δ*S*_r_^0^ were calculated using eq 3A,B, respectively
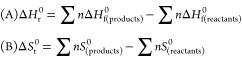
3where *n* is the stoichiometry.

Using the Gibbs equation, Δ*G*° was calculated at 298 K (eq 3)
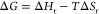
4Δ*G*° was then calculated for a temperature range of 40–160 °C relying on the Van’t Hoff approximation. Using eq 4, the equilibrium constant *K*_eq_ was calculated for each temperature as
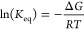
5Equation 5 was used to calculate product equilibrium concentrations from *K*_eq_
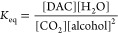
6A numerical optimization of eq 5 was implemented in python, and the code is freely available on GitHub.^54^

CO_2_ densities were obtained from Peace Software,^55^ and concentrations were determined calculated using the following equation
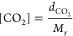
7where *d*_CO2_ is the CO_2_ density and *M*_r_ is the molecular mass of CO_2._

## Results and Discussion

### Catalyst Benchmarking with Dehydrating Agents

**Figure 1:**
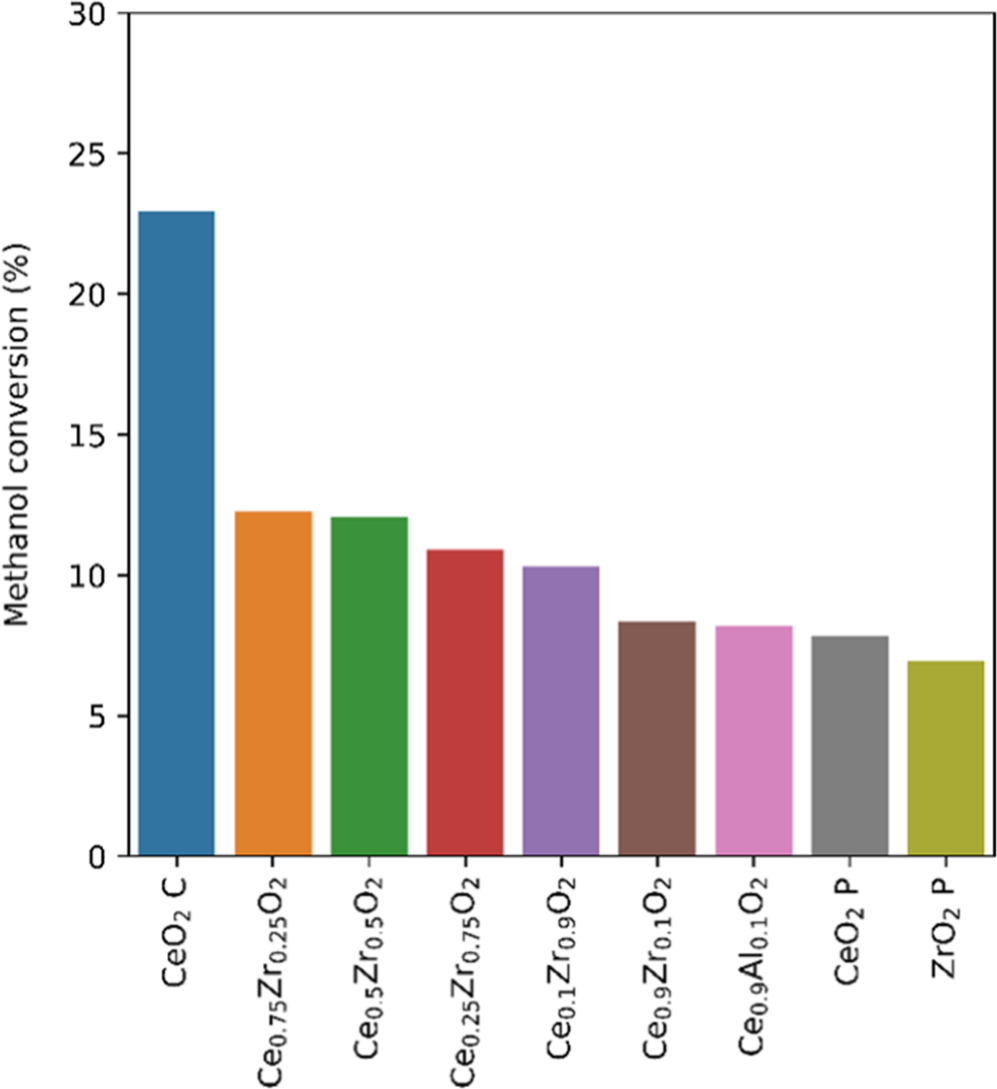
Comparison of metal oxide catalysts for effectiveness in catalytic DMC formation. Reaction conditions: 0.03 g of catalyst, 1 g of MeOH, 2:1 mol ratio of MeOH/DIC, pressurized to 50 bar CO_2_ at 40 °C prior to heating to 120 °C for 2 h (C = commerical, P = synthesized via precipitation).

As reaction conditions and catalyst performance metrics reported for the formation of DMC from CO_2_ vary in the literature, we started with a side-by-side comparison of some prominent catalyst materials^28,56^ to provide a basis for further investigations. This initial activity benchmarking was performed at 3 wt % catalyst loading in batch mode using diisopropyl carbodiimide (DIC) as a dehydrating agent at 120 °C. Figure 1 shows the level of MeOH conversion achieved by the various ceria and zirconia catalyst materials investigated (including a commercial CeO_2_ sample) after 2 h reaction time to gauge initial activity rather than final conversion.

**Figure 2:**
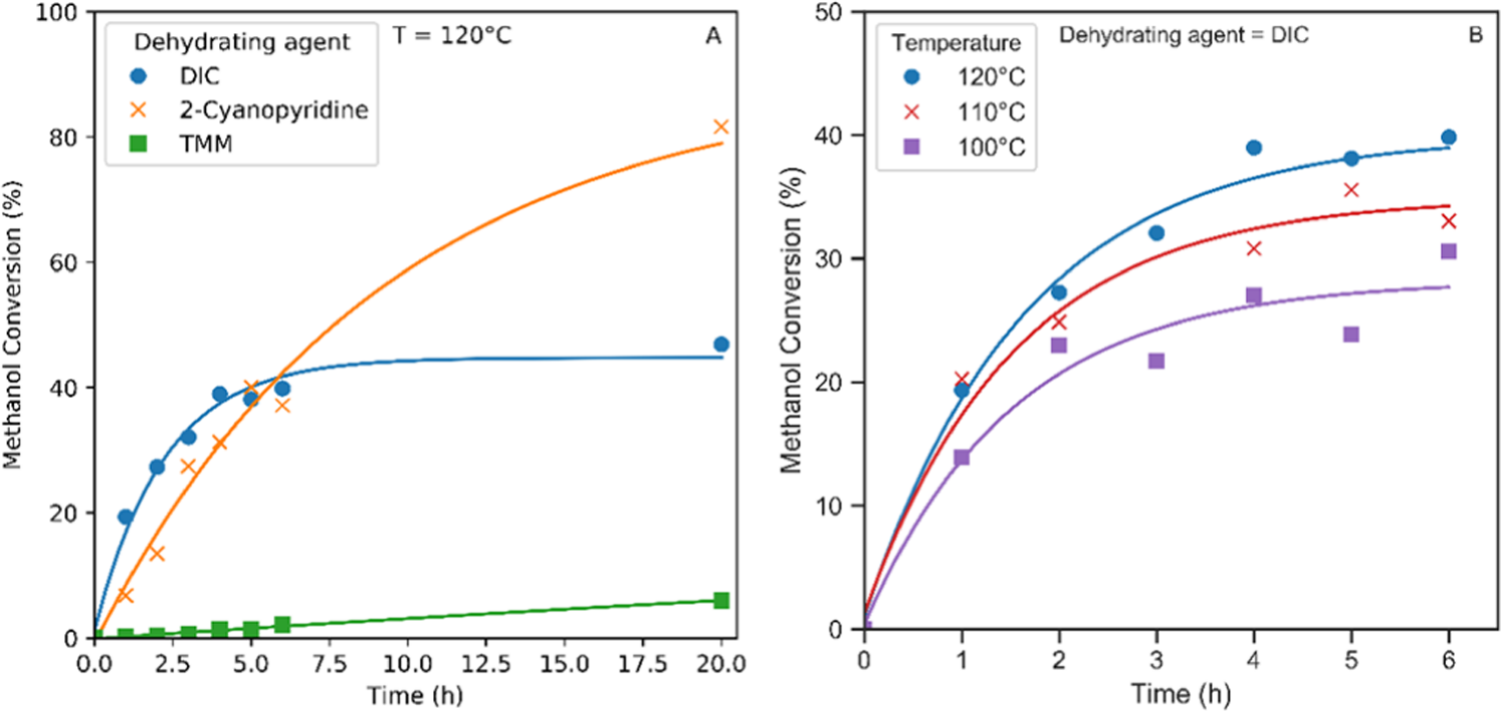
(A) CeO_2_-catalyzed MeOH conversion to DMC at 120 °C in the presence of different dehydrating agents. (B) CeO_2_-catalyzed MeOH conversion to DMC in the presence of DIC at different reaction temperatures (reaction conditions: 0.03 g of CeO_2_, 1 g of MeOH, 2:1 mol ratio of MeOH/dehydrating agent, pressurized to 50 bar CO_2_ at 40 °C prior to heating to reaction temperature).

All metal oxides synthesized via standard precipitation-calcination routes (for details, see the Supporting Information) gave conversions in the range of 7–13% after 2 h, with mixed Ce/Zr materials being slightly more effective than the pure oxides. This has previously been reported in DMC formation reactions without dehydrating agents and has been correlated to the favorable acid–base properties of the mixed metal oxides.^56−58^ A commercial CeO_2_ sample proved to be about twice as active as any other material tested, 23% compared to 12%, and was thus selected for all further experiments. This commercial sample had less than half of the BET surface area of the other catalysts used (Table S2), and it has been previously shown that factors such as calcination temperature, precursor, and surface acidity/basicity have more pronounced effects on activity in DAC synthesis than surface area.^33^ We briefly investigated its effectiveness with different dehydrating agents including the most commonly used diisopropyl carbodiimide (DIC), 2-cyanopyridine, and trimethoxy methane (TMM)^40,43,59^ in a 1:2 ratio with MeOH, giving a maximum achievable conversion of 100%. Figure 2A shows the profile of these reactions in batch mode sampled over time. At 120 °C, TMM achieved a conversion of only 3% after 6 h, which is greater than equilibrium with no dehydrating agent (see below) but much less than what was seen with the other reagents tested. As DMC yields of up to 33% have been reported under similar conditions with a 10 wt % loading of a mixed ceria/zirconia catalyst material supported on graphene,^40^ we suspect that the hydrolysis kinetics of TMM are unfavorable under our reaction conditions using the commercial CeO_2_ catalyst. Consistent with this assumption, other work successfully utilizing orthoesters used a more acidic catalyst for the orthoester hydrolysis to alcohols.^38,60^ 2-Cyanopyridine was more effective under our reaction conditions and gave a rather linear reaction profile, achieving >40% conversion after 6 h and continuing to produce 80% conversion after 20 h. With 15.6 mmol of 2-cyanopyridine used, the rates observed (78 mmol/g_cat_·h) are comparable to previous reports^30^ but about half of the highest activities reported.^29^ Like TMM, 2-cyanopyridine also requires an oxide catalyst to facilitate its reaction with water, which may lead to competition for active sites during the catalysis. Previous reports have also shown 2-cyanopyridine to be capable of promoting the reaction of an alcohol with CO_2_ in the absence of a catalyst,^44^ as well as assisting the formation of strongly basic sites, which can enhance the performance of the metal oxide catalyst.^61^ The activity shown is therefore likely to be a combination of the additional driving force provided by the dehydration as well as a kinetic acceleration of the reaction. DIC, which spontaneously reacts with water, gave the highest initial rates but began to plateau after 4 h and stalled at a maximum of 47% after 20 h. This behavior is ascribed to the precipitation of the corresponding urea that progressively coats the catalyst and noticeably thickens the reaction mixture (see Figure S1). The 104 mmol/g_cat_·h observed for DIC with commercial ceria compares favorably to other dehydrating agents used for this reaction.^62^

Using the effective CeO_2_-C material combined with DIC, we investigated the lower temperature limit of the catalysis in view of the unfavorable entropic term that bedevils the free condensation reaction without dehydrating agents. As shown in Figure 2B, the commercial CeO_2_ catalyst remained active down to 100 °C, with a decrease in both the initial rate and final conversion levels. The latter effect is ascribed to the lower solubility of the diisopropyl urea at lower temperatures, leading to a more pronounced inhibitory effect under reaction conditions. Temperatures below 100 °C gave much lower MeOH conversions (less than 10% after 3 h), showing 100 °C to be the onset temperature of catalytic activity of CeO_2_.

### Thermodynamics of the Free Condensation Reaction without Dehydrating Agents

As the use of dehydrating agents is clearly impractical for large-scale DAC production, we sought to apply the active CeO_2_ catalyst in the free condensation reaction under various conditions. To our surprise, we did not find precise equilibrium data for this widely studied reaction in the literature, so we set out to calculate equilibrium positions of the free condensation reaction over a range of reaction conditions (for details, see the Experimental Section). Standard heats of formation were available for all reagents and products, however with some variations depending on the source (Table S3). After testing a range of different scenarios and comparing them with experimental results (see below), we concluded the data in Table 1 to be the most accurate.

**Table 1:** Thermodynamic Values for Each Component Investigated in This Work at 298 K in Standard State

component	Δ*H*_f_^o^ (kJ/mol)	*S*^o^ (J/mol·K)	refs
MeOH_(l)_	–238.4	127.2	(63, 64)
EtOH_(l)_	–277.0	159.9	(63, 64)
CO_2(g)_	–393.5	213.8	(63, 64)
DMC_(l)_	–613.8	218.7	(13, 65)
DEC_(l)_	–681.6	293.3	(63, 65)
H_2_O_(l)_	–285.8	70.0	(63, 64)

**Scheme 2:**
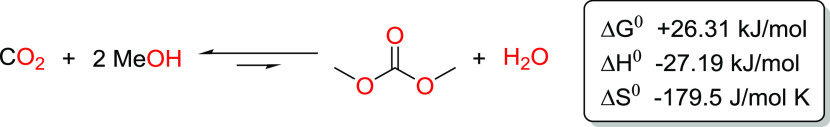
Reaction of CO_2_ and Methanol to Form DMC and Water, with Thermodynamic Data at 298 K

**Scheme 3:**
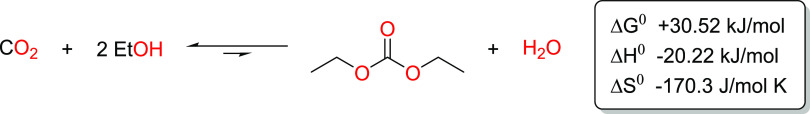
Reaction of CO_2_ and Ethanol to Form DEC and Water, with Thermodynamic Data at 298 K

Using these values, ΔH_r_ and ΔS_r_ can then be calculated for the formation of DMC and DEC, respectively (Schemes 2 and 3).

From these values, it can be seen that the formation of both DACs is exothermic (Δ*H*^0^ = −27.2 kJ/mol for DMC and −20.2 kJ/mol for DEC) along with a strongly endotropic term of −180 and −170 J/mol·K, respectively, making the reaction endergonic by more than 25 kJ/mol at room temperature already. Higher temperatures (as typically needed for catalysis to occur) will lead to even less favorable equilibrium positions. At a typical reaction temperature of 413 K, *K*_eq_ has a value of 1.16 × 10^–6^, which increases to 1.72 × 10^–6^ at 393 K, corresponding to DMC equilibrium concentrations of 55 and 85 mM, respectively.

For the larger ethanol molecule, the entropic change of the condensation reaction is slightly lower than that for methanol due to the higher number of degrees of freedom, but the enthalpic term for DEC formation is much lower than for DMC so that the Gibbs free energy for DEC formation is 4.2 kJ/mol higher than for DMC formation.

**Scheme 4:**
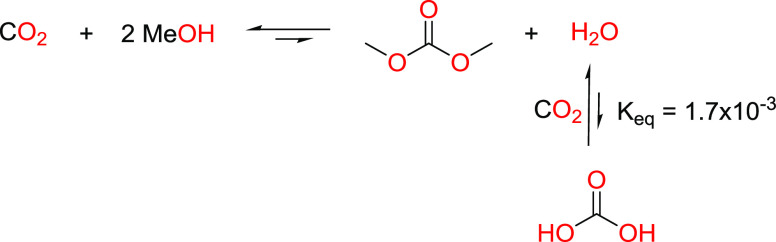
Reaction of CO_2_ and Water to Form Carbonic Acid, with Equilibrium Constant at 298 K^66^

We also considered the additional effect of the carbonic acid equilibrium that could potentially shift the DAC equilibrium position by consumption of water from the reaction mixture (Scheme 4). Due to the small equilibrium constant for the formation of carbonic acid, the latter has a negligible effect on the position of the DAC equilibrium (<0.01%) and is thus not considered in our following thermodynamic calculations. However, we note that the acidification of the reaction mixture, via both CO_2_ solvation (physisorption) and carbonic acid formation (chemisorption), may have kinetic implications for the catalysis such as exerting an accelerating effect for DAC formation or perhaps playing a role in catalyst deactivation pathways.

**Figure 3:**
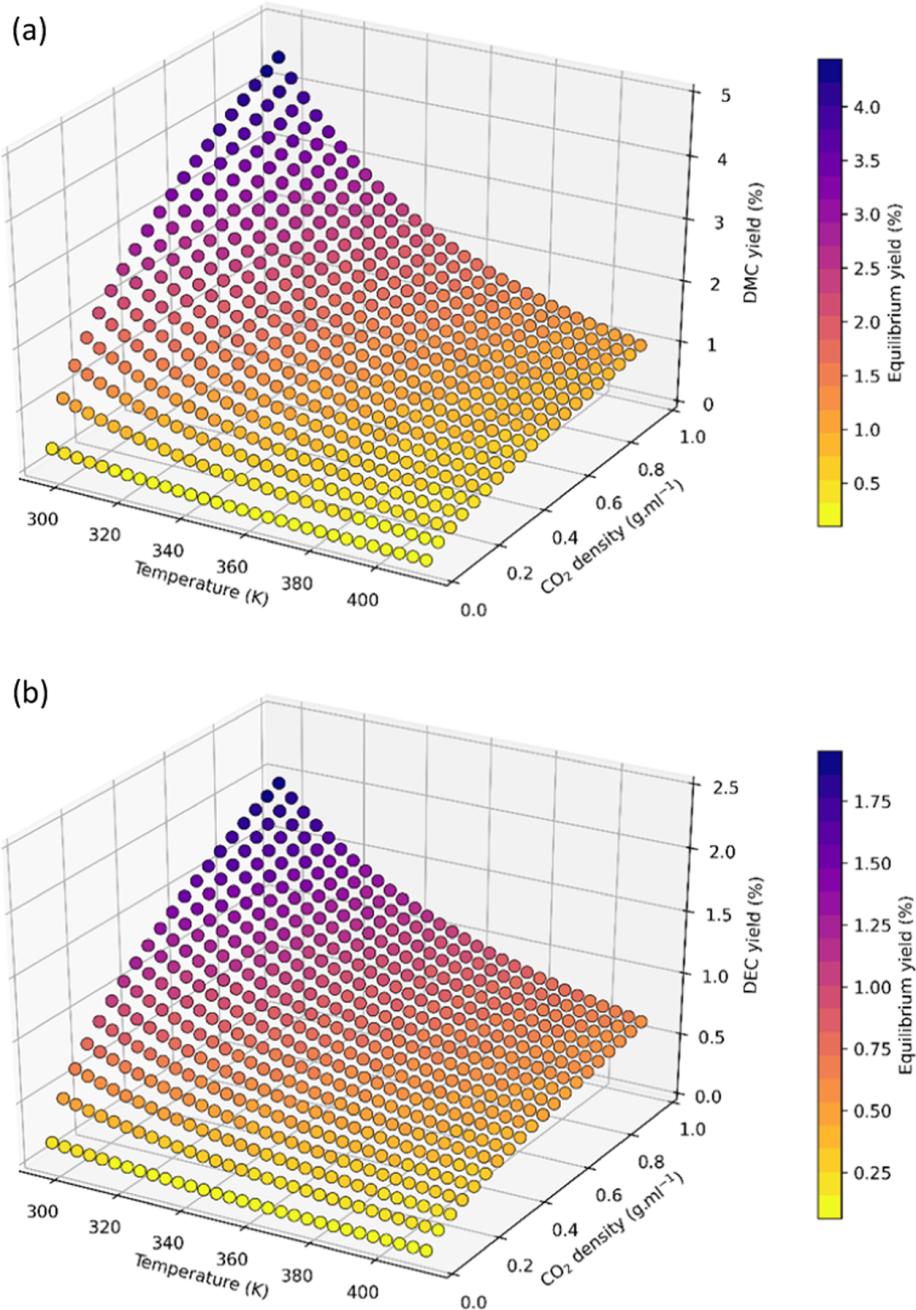
Calculated equilibrium conversions for the condensation of CO_2_ with (a) MeOH (Scheme 2) and (b) EtOH (Scheme 3) across multiple temperatures and CO_2_ densities.

Using the values shown in Table 1, a multivariate equilibrium calculation was carried out to translate the thermodynamic data into product yields over a range of reaction conditions (details can be found in the Supporting Information, including a link to the code used which is freely available). Figure 3a shows the equilibrium conversions of MeOH to DMC across a range of relevant temperatures and CO_2_ densities in a closed system. As expected from the negative reaction entropy term, DMC yield decreased with increasing temperature. Increasing CO_2_ density (pressure) increased the conversion of MeOH to DMC due to higher CO_2_ concentrations. The model showed that DMC yields up to 4.5% are possible in liquid CO_2_ at room temperature (56 bar), whereas only about 1% conversion can be achieved at more typical reaction temperatures of 100–140 °C where CO_2_ densities are much lower, even when going to very high pressures of 1000 bar. For EtOH conversion to DEC a similar trend was seen (Figure 3b), but due to its lower Δ*G*_r_, DEC equilibrium yields were about half of those of DMC under the same conditions, giving a maximum DEC yield of 2% in liquid CO_2_ at room temperature which declines to about 0.6% at higher reaction temperatures.

The model can also be used for determining what effect residual moisture in the starting materials has on the equilibrium conversion of the alcohol (Figure S5). This allows for the calculation of upper and lower limits of the equilibrium conversions given a range of moisture expected within the starting materials used. Most importantly though, these thermodynamic limits under the reaction conditions applied allow assessing the effectiveness of catalytic reactions without dehydrating agents in terms of absolute rates, conversion levels, and space-time yields rather than the often-used normalized metrics of activity per catalyst mass or surface area.

### Catalytic Batch Reactions without Dehydrating Agents

**Figure 4:**
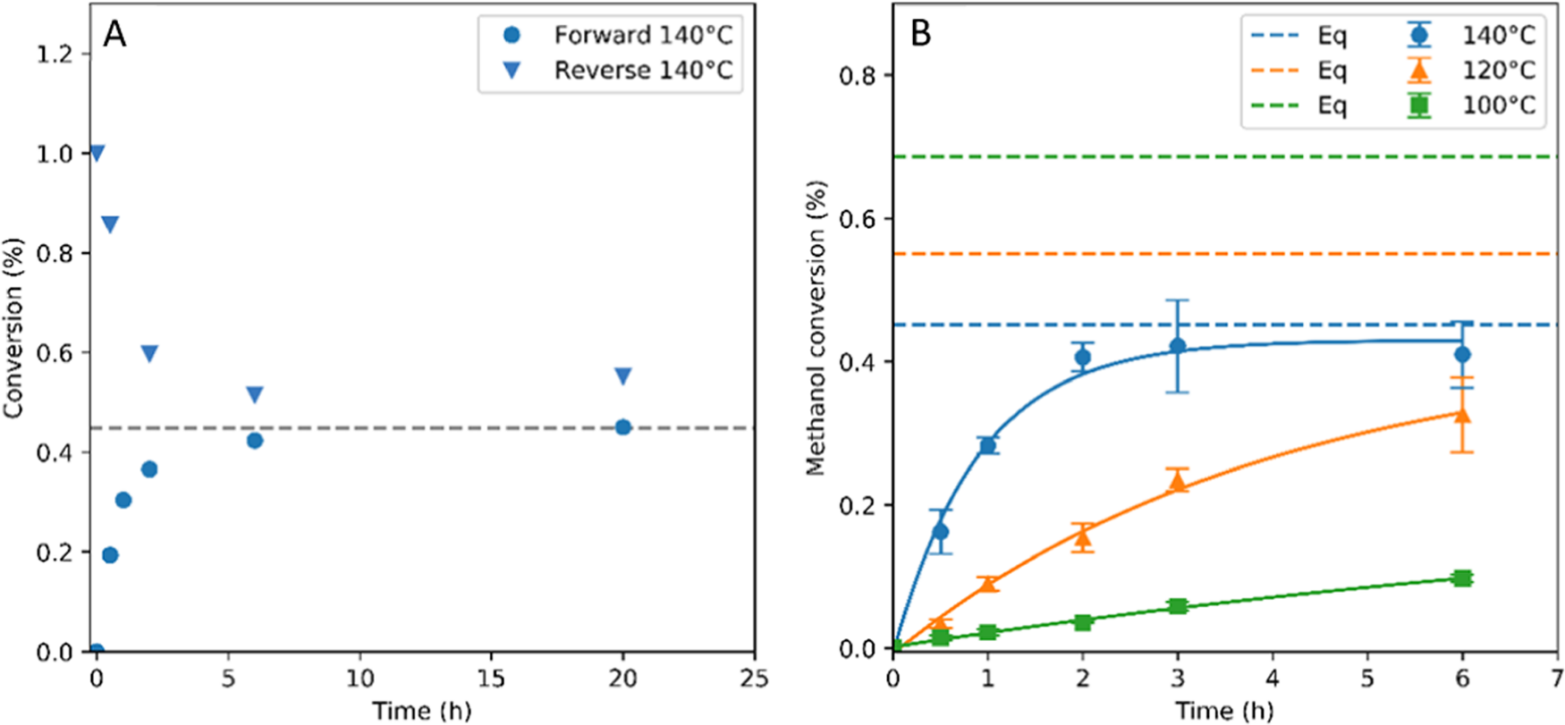
(A) DMC formation from MeOH and CO_2_ catalyzed by CeO_2_ (forward reaction) as well as DMC hydrolysis from twice the equilibrium conversion (reverse reaction) at 140 °C. (B) DMC formation from MeOH and CO_2_ catalyzed by CeO_2_ at different temperatures over time. Dashed lines indicate calculated equilibrium conversions, with [MeOH] = 24.7 M and [CO_2_] = 4.5 M. Reaction conditions: 0.3 g of CeO_2_, 70 bar CO_2_ at 40 °C (0.2 g/mL), 5 mL of dry MeOH (errors derived from triplicates).

**Figure 5:**
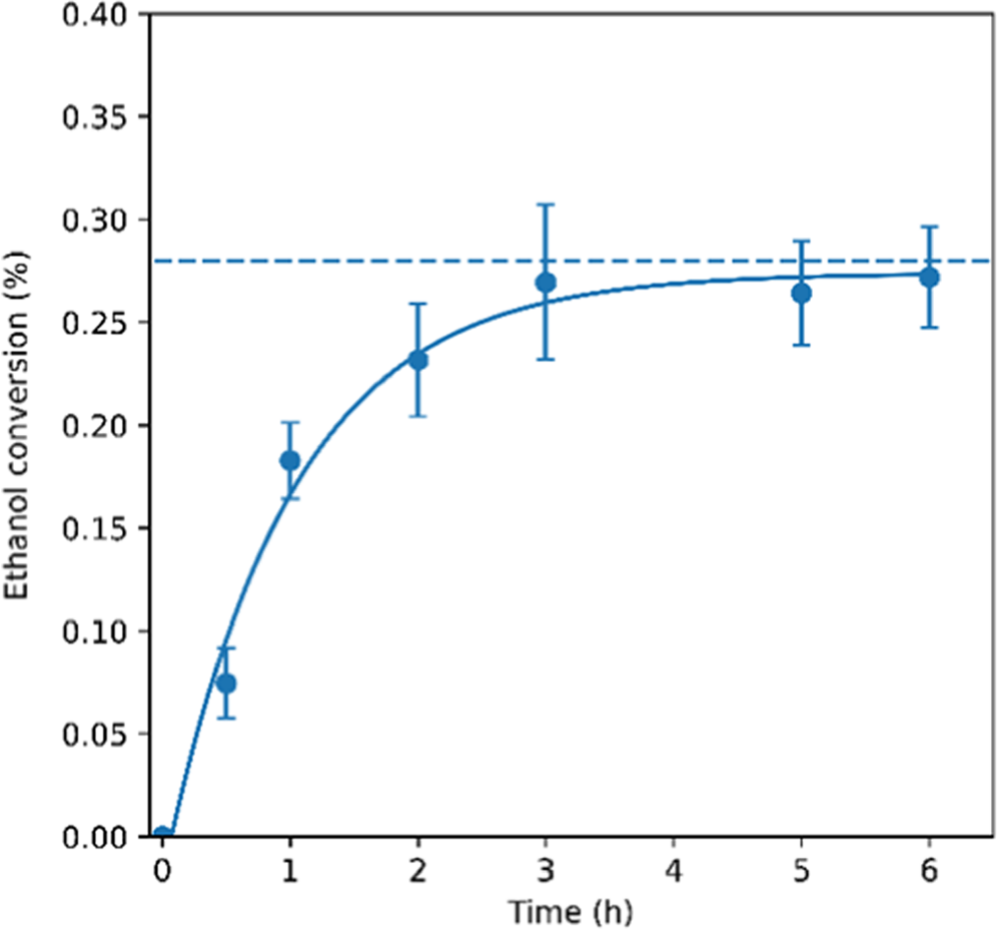
DEC formation from EtOH and CO_2_ catalyzed by CeO_2_. Dashed lines indicate calculated equilibrium conversion, with [EtOH] = 17.1 M and [CO_2_] = 4.5 M. Reaction conditions: 0.3 g of CeO_2_, 70 bar CO_2_ at 40 °C (0.2 g/mL), 5 mL of dry EtOH, 140 °C (errors derived from triplicates).

With this information in hand, the direct synthesis of DMC from MeOH and CO_2_ over a few CeO_2_-based catalysts was assessed, without any dehydrating agents, in a batch reactor. Following some initial catalyst testing (Table S1), the commercial CeO_2_ catalyst was selected for further reactions. Both the forward reaction to give DMC and the reverse reaction (hydrolysis of DMC back to MeOH and CO_2_) were investigated to test the accuracy of our equilibrium calculations. Figure 4a shows that the commercial CeO_2_ catalyst was active at 140 °C, with MeOH conversion progressing rapidly over the first 2 h with an initial rate of approximately 58.4 mM/h to converge to 0.4% DMC yield after about 6 h. The reverse reaction starting with 1% water and DMC mixture converged on the calculated equilibrium with an initial rate of approximately 64.8 mM/h, plateauing at 0.55% MeOH conversion (reverse reactions at 120 and 100 °C are shown in Figure S13). The equilibrium value for MeOH conversion under these conditions predicted by the above calculations was 0.45%, showing the model to be valid and indicating that the observed plateau was indeed caused by the system reaching equilibrium. When the reaction temperature was lowered to 120 °C, the initial rate decreased to 10.8 mM/h (Figure 4b), and a conversion of 0.32% was reached after 6 h. Decreasing the reaction temperature further to 100 °C gave a much lower initial rate of 2.24 mM/h, and the CeO_2_ catalyst only achieved 0.1% MeOH conversion after 6 h. By 30 and 20 h, respectively, reactions at 120 and 140 °C had reached their predicted equilibrium conversions. By 30 h at 100 °C, only 56% of the calculated equilibrium had been reached (MeOH conversion of 0.38%) due to the consistently lower rate at this temperature. For the reaction of EtOH with CO_2_ to give DEC at 140 °C, the initial rate of reaction was approximately 21.7 mM/h, with the conversion profile approaching the calculated equilibrium position of 0.29% after 6 h (Figure 5).

### Hydrolytic Stability of DMC

It has been previously observed in DMC synthesis that the product readily hydrolyzes back to CO_2_ and methanol,^67^ and our above experiments (Figure 4a) indeed showed the CeO_2_ catalyst to be equally active in the forward and backward reactions. This potentially presents a challenge for *in situ* separation strategies targeted at increasing productivity by continually removing the product from the reactor. With a view to continuous flow operation with integrated downstream separations, we investigated the stability of aqueous DMC solutions in the absence of CeO_2_. When the 1% DMC/water/MeOH mixture used for the catalytic reverse reaction shown in Figure 4a was heated to 140 °C in the absence of a catalyst, less than 10% hydrolysis of DMC back to MeOH and CO_2_ occurred over 20 h, indicating promising stability of DMC in the presence of moisture. A more detailed investigation following DMC hydrolysis at lower temperatures over longer timescales (Figure S14) yielded surprisingly long half-lives of DMC even under mildly acidic conditions (Table 2 and Scheme 4). A solution pH of 3 was used for these experiments as this has previously been determined to be the lower limit of aqueous solutions under 100 bar CO_2_.^68^

**Table 2:** Rate of DMC Hydrolysis in Aqueous Solution^a^

temperature (°C)	rate (mol/L·h)	*t*_1/2_ (days)
80	3.94 × 10^–4^	275
100	5.85 × 10^–4^	185

a Conditions: 1 mL of DMC (5.2 M), 0.236 mL of H2O (acidified to pH 3 with HNO_3_) in DMSO, analyzed by quantitative ^1^H NMR in a sealed J-Young tube.

At a 5.2 M concentration with equimolar quantities of water, the half-life of DMC was found to be ∼9 months at 80 °C and ∼6 months at 100 °C assuming a zero-order hydrolysis reaction (Figure S12). Applying the same rates to more typical reaction conditions of 87 mM DMC, we calculated zero-order *t*_1/2_ for DMC to be 4 and 3 days at 80 and 100 °C, respectively. These results show the product mixture to be quite stable even at reaction temperature when no longer in contact with the catalyst, opening the door for *in situ* enrichment strategies that increase yields beyond the values calculated in the homogeneous equilibrium system shown in Figure 3.

### Catalytic Continuous Flow Reactions without Dehydrating Agents

While batch reactions enable convenient screening for activity and scoping of reaction parameters,^13,30,34,56,69^ they typically have lower productivity than continuous flow processes due to nonproductive downtimes associated with charging/cleaning, heating/cooling, etc.^24,70^ The tight control of reaction conditions in a steady-state continuous flow reactor is another advantage over batch testing that may be exploited to accurately screen a range of reaction conditions on the same catalyst bed.^24^ Finally, catalyst stability tests in continuous flow are more realistic than batchwise recycling experiments that may be influenced by the repeated change in conditions. For the synthesis of DACs from short-chain alcohols and CO_2_, the use of compressed (near- or supercritical) CO_2_ as the carrier in a flow system is particularly appealing, as such media are known to combine gas-like diffusivity with solution-like solubility.^71^ In addition, the option of tunable phase behavior by modulation of temperature and pressure offers interesting possibilities for separations of the product mixture obtained.^72−74^ We thus investigated the performance of the CeO_2_ catalyst for DAC formation as a fixed bed in a continuous flow reactor^53^ using neat, dry alcohol dissolved in scCO_2_ (for experimental details, see the Supporting Information).

**Figure 6:**
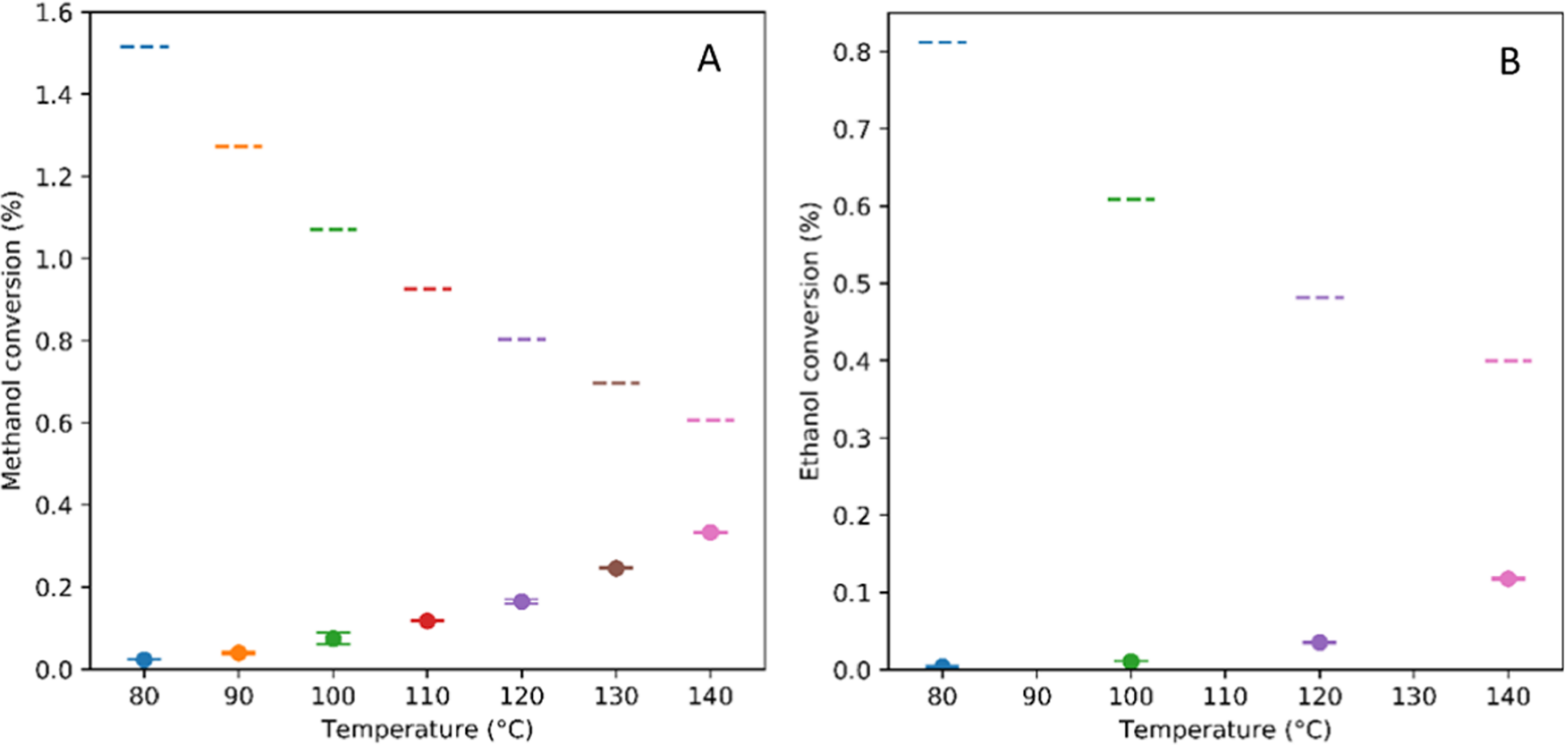
Steady-state alcohol conversions (A = MeOH, B = EtOH) to DAC in continuous flow mode with CO_2_ over a fixed bed of CeO_2_ at different temperatures (dots) including calculated equilibrium values (dashed lines). Reaction conditions: 1 mL/min CO_2_, 200 bar, 0.2 mL/min alcohol, 0.3 g of catalyst, 5 mL of bed volume, contact time 4.2 min.

When the temperature was varied from 80 to 140 °C at 200 bar and a constant flow rate of CO_2_ through a fixed bed of CeO_2_ at a contact time of 4.2 min, steady-state conversions increased progressively for both MeOH and EtOH due to the activity of the catalyst increasing with temperature (Figure 6). As expected from the thermodynamics, equilibrium conversions calculated under these conditions followed the opposite trend due to the enthalpy of the reaction (Figure 3) as seen before in batch (Figure 4B).

While the activity of CeO_2_ for DAC formation was low below 100 °C, DMC yields of >50% of the equilibrium value could be obtained at 140 °C with a contact time of less than 5 min. As seen in batch before, DEC formation levels were about half those of DMC across all temperatures. Comparing rates and productivities of batch versus flow experiments under consideration of the different reaction conditions (Table 3), we can see that DMC formation is about 8 times more efficient in flow (entries 7 and 3) and DEC formation about 7 times more efficient in flow than in batch (entries 8 and 4), notably all with the same CeO_2_ catalyst.

**Table 3:** Comparison of Productivity Metrics between Batch and Flow Experiments

entry	reaction	temperature (°C)	substrate/cat ratio (mol/mol)	observed rate (mM/h)	normalized rate (mM/h·g_cat_)	TOF (h^–1^)^c^	productivity (mmol/(L·h)
1	DMC batch	100	71	2^a^	7	0.020	0.89
2	DMC batch	120	71	11^a^	36	0.063	2.76
3	DMC batch	140	71	58^a^	195	0.231	10.1
4	DEC batch	140	49	27^a^	72	0.436	3.78
5	DMC flow	100	12	87^b^	290	0.059	18.6
6	DMC flow	120	12	205^b^	683	0.125	39.3
7	DMC flow	140	12	417^b^	1390	0.279	87.6
8	DEC flow	140	8	193^b^	644	0.100	20.5

a Observed rate calculated from tangent at *t* = 0 of the fitted curve.

b Observed rate calculated as concentration change in 4.2 min residence time.

c TOF calculated from total moles of product formed/total moles of catalyst per time.

**Figure 7:**
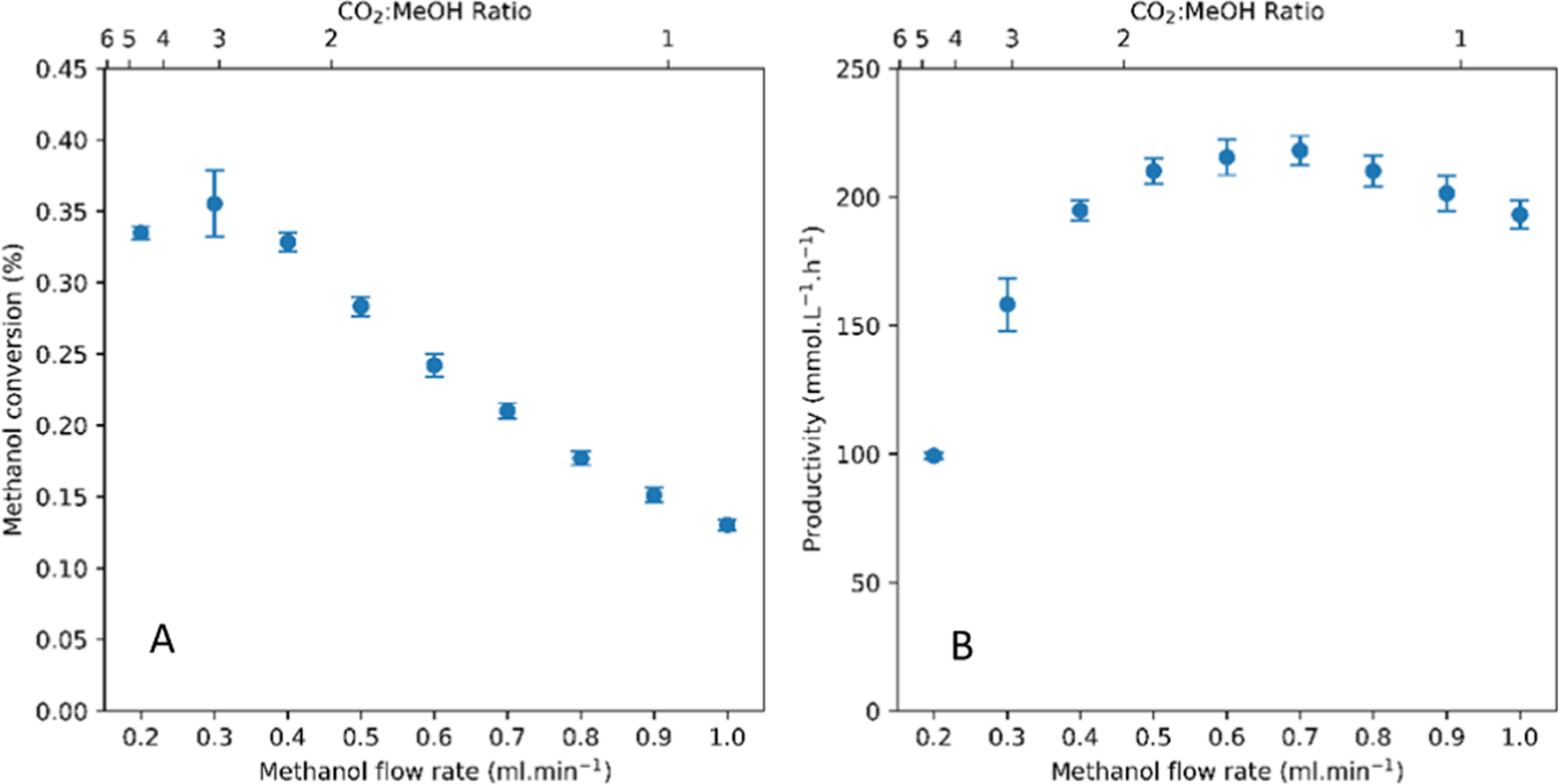
MeOH conversion at various feed rates (A) and corresponding DMC productivity values (B). Reaction conditions: 140 °C, 1 mL/min CO_2_, 200 bar, 3 g CeO_2_, 5 mL bed volume.

When the methanol feed rate was increased at constant CO_2_ flow and pressure at 140 °C, DMC conversions decreased progressively further away from the equilibrium value of 0.6% (Figure 7). Note that the higher amount of substrate did not significantly alter the total flow rate (and thereby contact time with the catalyst) due to it remaining a single phase,^75^ but merely increased MeOH concentration in the scCO_2_.

Although the percentages of substrate converted decreased with increasing MeOH content in the feed, the absolute number of moles of substrate converted increased, producing more DMC over the same period. Plotting DMC productivity of the system over MeOH flow rate indeed showed a maximum DMC output of 235 mmol/L·h at intermediate feed rates of around 0.5 mL/min MeOH (at 140 °C and 200 bar CO_2_). This value is among the highest DMC productivity for the direct catalytic condensation of MeOH with CO_2_ (without water removal) reported to date.^18^ The conditions giving maximum productivity were found at a 1.5:1 ratio of CO_2_ to MeOH at 25% reaction progress in steady state (0.2% conversion vs. 0.8% equilibrium), which appeared to be the optimum balance of rate and conversion level that resulted in maximum productivity per unit time. Increasing the CO_2_ flow rate at 1 mL/min MeOH feed decreased the catalyst contact time without increasing the number of moles converted, leading to both lower conversions and lower DMC productivities (Figure S7).

**Figure 8:**
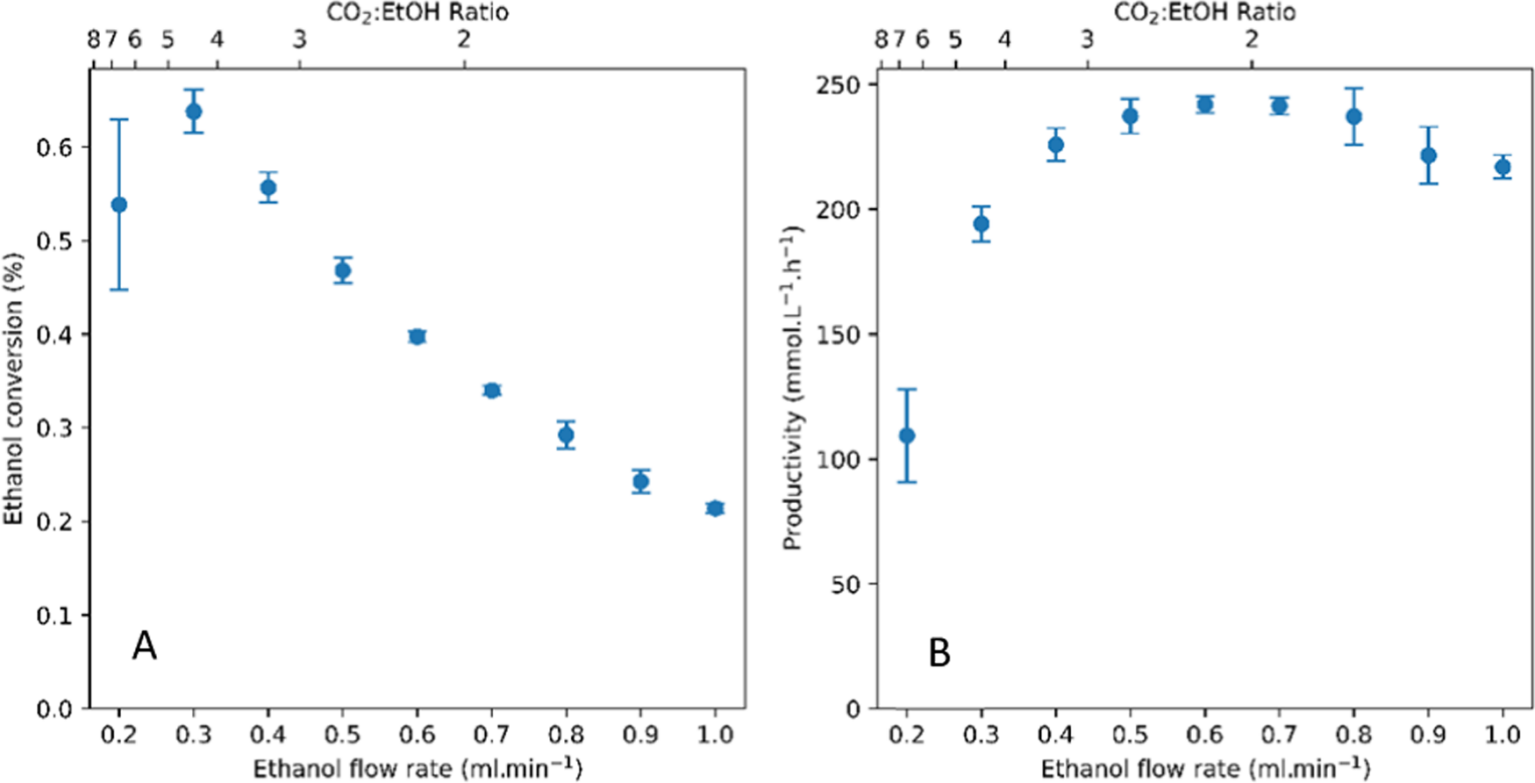
EtOH conversion at various feed rates (A) and corresponding DEC productivity values (B). Reaction conditions: 140 °C, 1 mL/min CO_2_, 200 bar, 3 g CeO_2_, 5 mL bed volume.

Performing the same feed ratio variation in continuous flow with EtOH gave similar results (Figure 8). At 140 °C and 200 bar, we found peak productivity of the catalytic process at ∼50% steady-state reaction progress (0.4% conversion vs. 0.8% equilibrium) around a 2:1 CO_2_ to EtOH ratio as the optimum compromise between rate and conversion. Again, the DEC space-time yield of 240 mmol/L·h achieved under these conditions is one of the highest values reported to date.^24,76^

### Catalyst Stability in Continuous Flow

While having an active catalyst and productive reactor system is important, stability of the catalyst is a key criterion for industrial application. One of the advantages of using a flow system is that it negates the need for catalyst recovery between batchwise recycling experiments and allows for a more meaningful assessment of its intrinsic stability toward sustained reaction conditions.^77^ In previous work, different types of CeO_2_ have been investigated for their stability in the reaction of MeOH with CO_2_ under various conditions, and several studies have reported modification procedures to combat some of the deactivation seen in repetitive batch experiments with different types of ceria.^28^ As good activity and no obvious signs of deactivation were observed in our initial experiments, the long-term stability of commercial, unmodified CeO_2_ was assessed for the condensation of MeOH and EtOH with CO_2_ in continuous flow mode at 140 °C and 200 bar, respectively.

**Figure 9:**
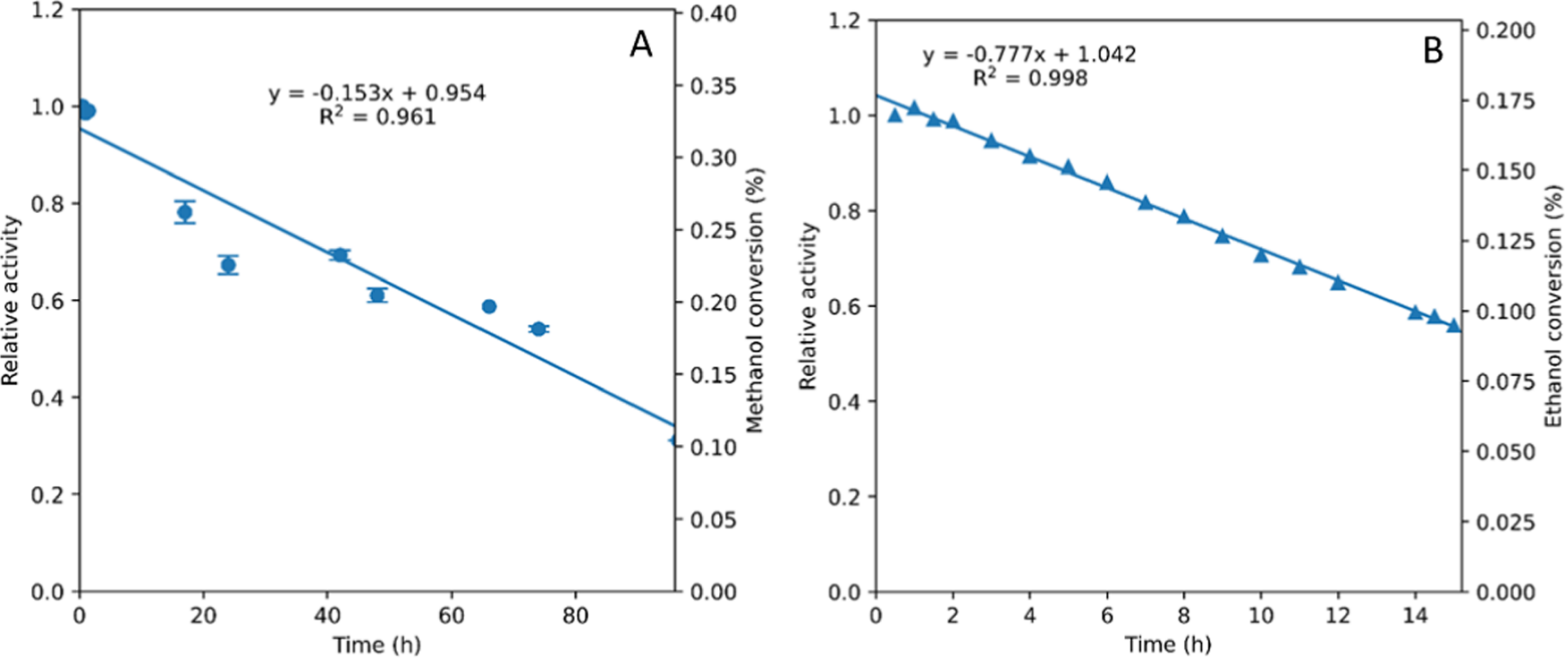
Stability of commercial CeO_2_ in catalytic DAC formation in continuous flow (A = MeOH, B = EtOH). Reaction conditions: 0.2 mL/min alcohol, 1 mL/min CO_2_, 200 bar, 140 °C, 0.3 g CeO_2_, contact time 4.2 min.

Figure 9 shows the formation of DMC (A) and DEC (B) over extended times on stream with no change in reaction conditions. In the methanol reaction, the catalyst lost 68% of its activity over the course of 96 h, corresponding to a zero-order *k*_d(obs)_ of 0.15/day, or a catalyst half-life of 6.6 days. The reaction of ethanol with CO_2_ caused a more pronounced deactivation, with the catalyst losing around 44% of its activity over 15 h corresponding to a *k*_d(obs)_ of 0.77/day or a catalyst half-life of 1.3 days. This corresponds to a 5 times faster deactivation with EtOH than with MeOH. Attempts to regenerate the catalyst by heating the bed to 120 °C under reduced pressure failed to restore activity.

**Figure 10:**
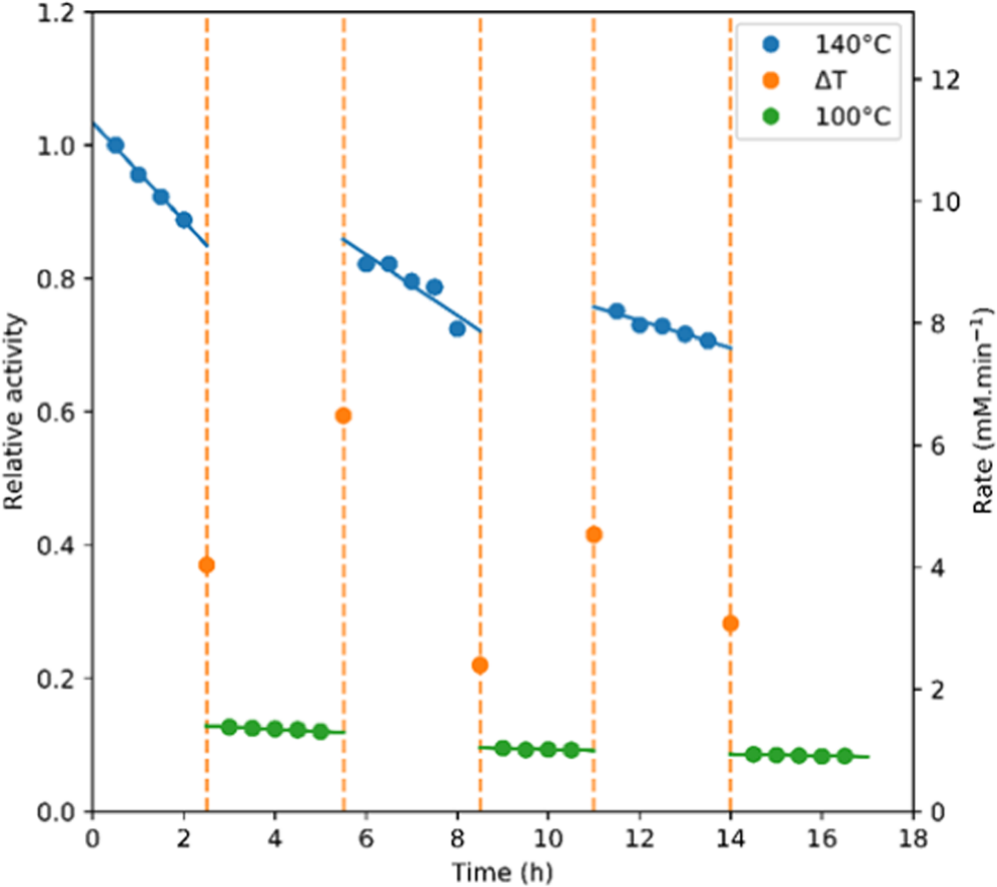
Relative activity of commercial cerium oxide when cycled between 140 and 100°C; dashed lines indicate the 30 min for temperature stabilization. Conditions: 0.2 mL/min MeOH, 1 mL/min CO_2_ at 200 bar, 0.3 g of CeO_2_, contact time 4.2 min.

To gain more insight into the deactivation behavior of CeO_2_ under sustained reaction conditions in continuous flow, accelerated aging experiments were carried out with MeOH. Repeatedly cycling the catalyst bed between 140 and 100 °C (Figure 10) showed CeO_2_ deactivation to be about 16 times slower at 100 °C. The initial *k*_d(obs)_ observed were larger than those derived from the long-term experiments shown in Figure 9 but decreased with each cycle, suggesting the most active sites to quickly deactivate before slower progressive catalyst deactivation takes over linearly (Table 4).

**Table 4:** Deactivation Rates at Each Temperature and Cycle for Commercial Cerium Oxide

temperature cycle	140 °C *k*_d(obs)_ (d^–1^)	100 °C *k*_d(obs)_ (d^–1^)
1	–1.77	–0.078
2	–1.10	–0.040
3	–0.49	–0.031

**Figure 11:**
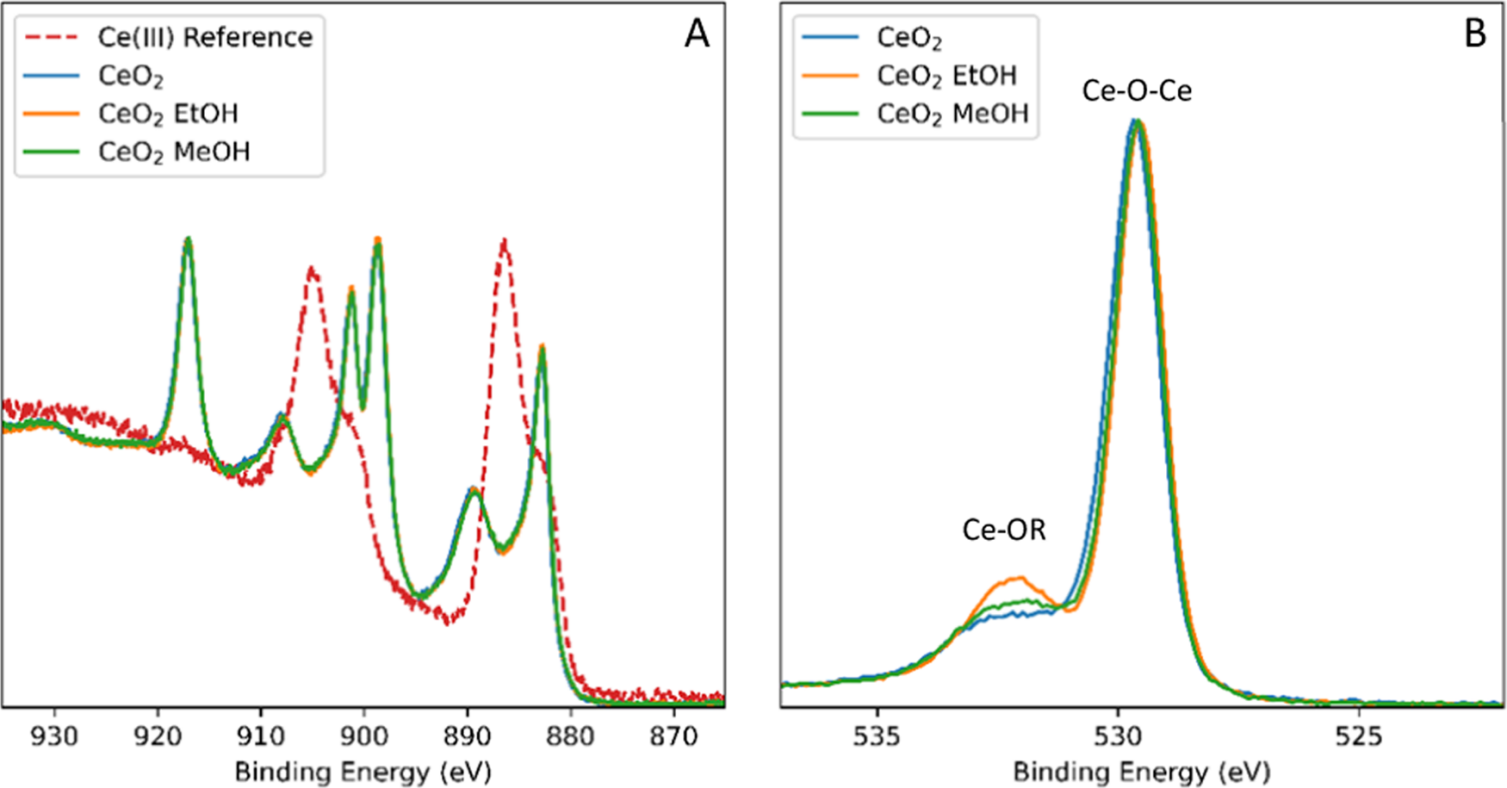
XPS spectra of unused catalyst (CeO_2_) and following a 6 h reaction with ethanol and methanol: (A) cerium region with a Ce(III) reference and (B) oxygen region.

Aresta proposed the main mode of catalyst deactivation for ceria in catalytic DMC formation to be a progressive reduction of active Ce(IV) sites to inactive Ce(III).^28^ Analyzing our partially deactivated CeO_2_ by X-ray photoelectron spectroscopy after conversion had just begun to decrease after a short amount of time on stream showed no discernible levels of reduction to Ce(III) (Figure 11). Instead, an increased level of oxygen defects was observed in the material post-reaction that is indicative of a decrease in the number of O^2–^ sites on the surface of the material. The anionic defect sites thus created may be occupied by hydroxides, alkoxides, or carboxylates, implying the first step leading to catalyst deactivation to involve a loss of basic Ce–O–Ce sites that are required for CO_2_ binding and activation.^20,35^ This is most likely caused by hydrolysis to acidic Ce–OH hydroxyls (possibly facilitated by carbonic acid formed *in situ*), Ce-OR alkoxides, or Ce-OOR carboxylates that are difficult to regenerate to Ce–O–Ce sites required for catalytic DAC formation. As significant degrees of reduction to Ce(III) have been detected in CeO_2_ catalysts by XPS after more pronounced deactivation,^28,36^ the hydrolysis of surface Ce–O–Ce units observed here after a low number of catalytic turnovers likely represents an entry point into catalyst deactivation that subsequently entails irreversible cerium reduction. Ethanol being more reducing than MeOH ultimately leads to faster catalyst deactivation, and more robust catalyst materials will be needed for DEC synthesis in particular.

### Effect of Product Removal

A number of publications have pursued strategies to remove water from the reaction via nonreactive (physical) separation strategies to shift the unfavorable equilibrium position of DAC formation from alcohol and CO_2_.^24,76,78^ While this represents a more realistic approach than adding synthetic, high-energy water scavengers that change the thermodynamics of the reaction (see above), these approaches have met with limited success so far. Typically, absorber beds are added as a separate unit downstream of the catalyst bed, and the dried DMC/MeOH/CO_2_ mixture fed back into the reactor to re-establish equilibrium.

**Figure 12:**
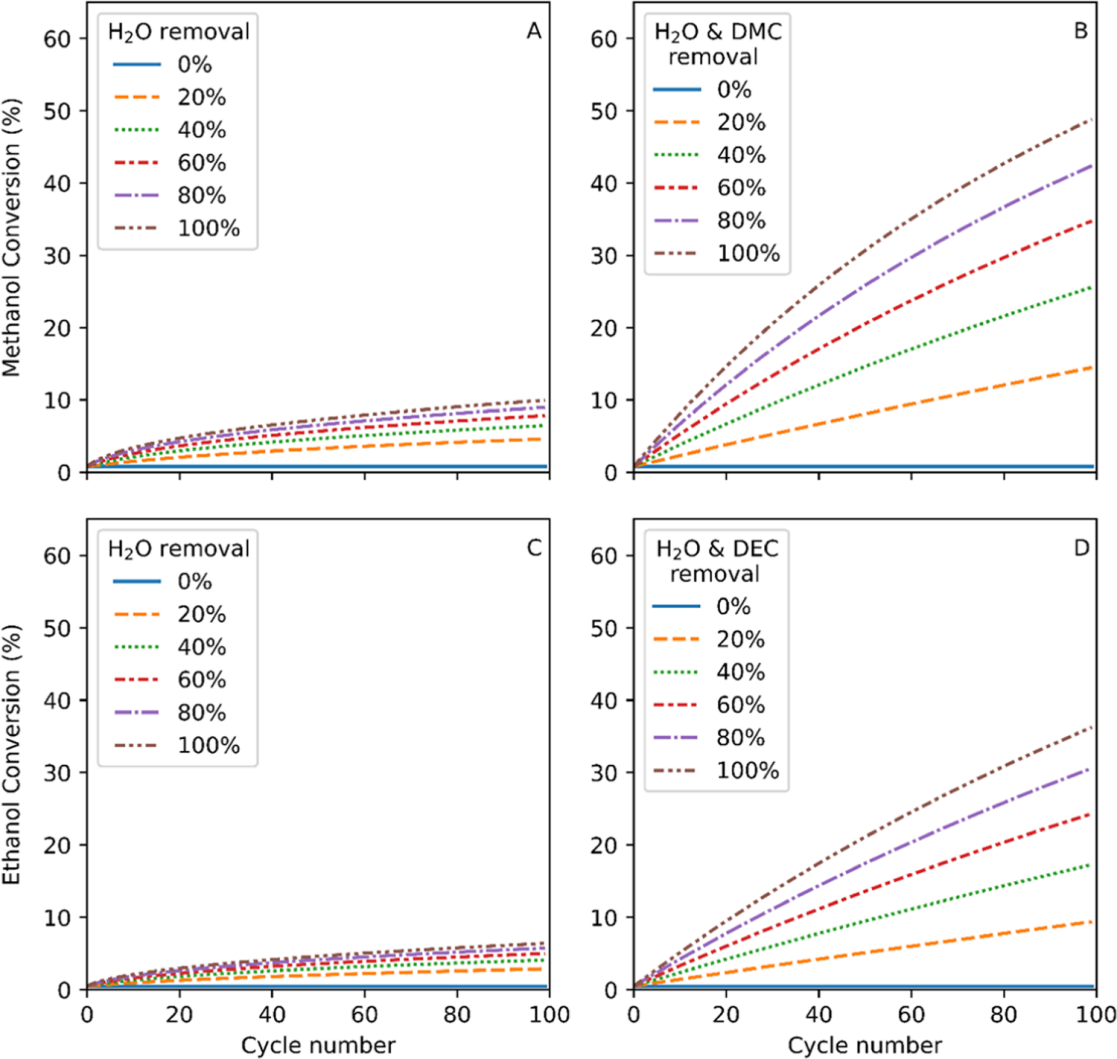
Calculated methanol and ethanol yields with iterative product removal at various drying efficiencies. (A) Cumulative DMC yields with water removal. (B) Cumulative DMC yields with equal water and DMC removal. (C) Cumulative DEC yields with water removal. (D) Cumulative DEC yields with equal water and DEC removal. Model parameters: 140 °C, 0.358 g/mL CO_2_ (200 bar). Starting concentrations: 13.36 M CO_2_, 10.19 M MeOH, 7.05 M EtOH, 0 M DAC, and 0 M H_2_O.

With our thermodynamic model we can calculate the efficiency of such approaches that iteratively shift the equilibrium position to higher DAC concentrations by stepwise removal of water from the mixture.^a^Figure 12 shows a thermodynamic prediction of how effective these stepwise, nonreactive water removal strategies may be. This is achieved by calculating the initial equilibrium position of the alcohol to DAC reaction as above (in this case at 140 °C and 200 bar) and then removing a percentage of the H_2_O byproduct from the mixture. The system is then allowed to re-equilibrate via repeated contact with the catalyst and the cycle repeated. The varying percentages of water removal reflect different degrees of drying efficiency, with a theoretical 100% efficiency representing the thermodynamic best-case limit.

For the MeOH to DMC reaction (Figure 12a), we can see that 100 cycles with complete water removal would allow us to accumulate a DMC yield of around 7%. Previously, Choi et al.^25^ reported >40% DMC yield using a loop reactor including a drying column packed with 3 Å molecular sieves under similar (supercritical) flow conditions. We calculate that over 2000 drying cycles at 100% efficiency are necessary to achieve this result, and the system reported indeed required 15 g of drying agent to push 3.2 g of MeOH to 40% DMC conversion over the course of three days (corresponding to a peak productivity of 90 mmol/L·h, decreasing to 16 mmol/L·h after 70 h).

For the equivalent EtOH-to-DEC reaction, we find similar results (Figure 11c), with 100 cycles of perfect drying efficiency increasing the yield from 5 to 38%. Separation of water and DEC using membranes has recently been demonstrated for DEC synthesis by Wang et al.*,*^24^ where a 30% enrichment of DEC and water was observed in their permeate at 0.02% conversion. Similarly, Dibenedetto et al.^76^ demonstrate an improvement in ethanol conversion from 0.9% up to 3% with pervaporation membranes.

These results show that water removal alone is unlikely to improve the efficiency of DAC production from alcohols and CO_2_ to the point where it may become economically viable, as either marginal gains in yield are obtained or large amounts of drying agent and cycle numbers are required. From a process perspective, it is thus worth considering alternative separation schemes.

**Figure 13:**
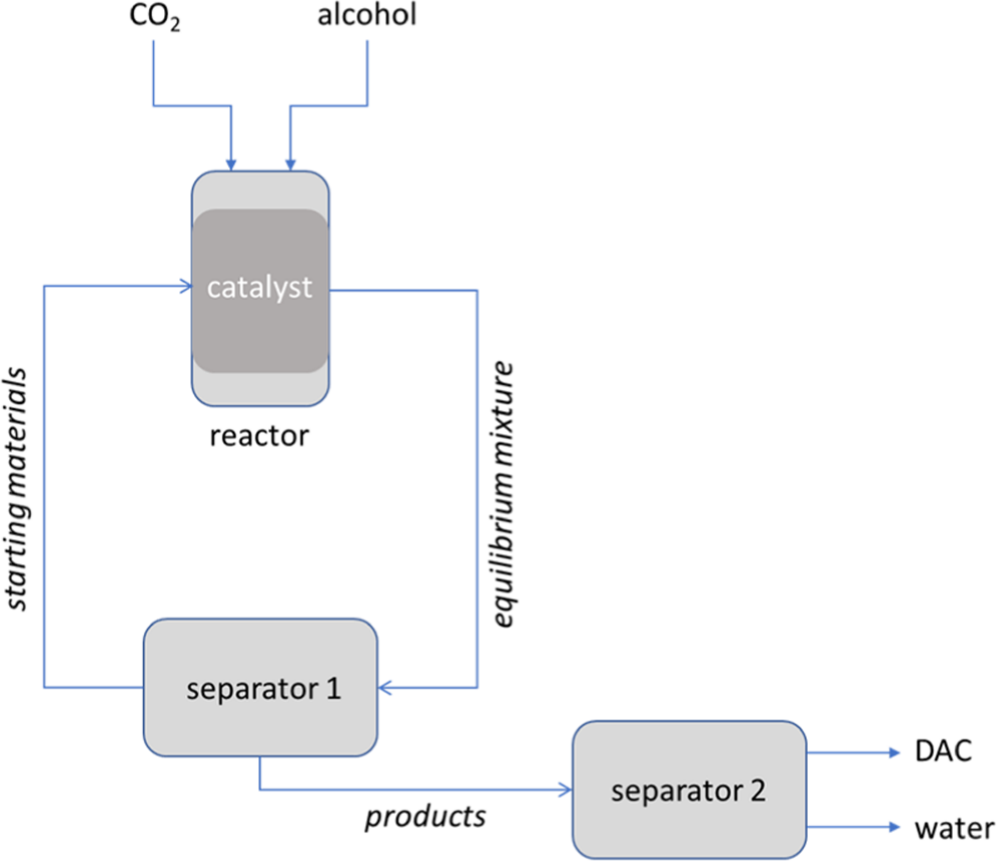
Continuous flow process schematic with the DAC reactor coupled to a series of two separation units (pumps and process controls not shown). Liquid CO_2_ and alcohol are equilibrated with DAC and water over a packed catalyst bed in the reactor, and the pressurized mixture passed through a separator that removes the product mixture (water + DAC). Unconverted starting materials (CO_2_ and alcohol) are recycled back into the reactor where they are continuously topped up with fresh reagents. The liquid DAC/H_2_O product mixture (which is stable in the absence of a catalyst) is further separated by fractionation. Segregating all three units would allow for optimum conditions to be applied at each operation (high *T* and high *p* in the reactor, low *T* and high *p* in separator 1, low *p* and high *T* in separator 2).

The removal, recovery, and reuse of excess CO_2_ from the reaction is straightforward due to it forming a separate gas phase upon depressurization. The remaining liquid phase composed of excess alcohol with <1% water and DAC is stable in the absence of a catalyst (see above) but difficult to purify due to the low product concentration and azeotrope formation. It has previously been reported that a complete separation of this ternary mixture by way of fractional distillation becomes economically viable from DAC contents of >6%.^79^ An alternative approach would be to only recover unreacted alcohol and CO_2_ in the first instance, recycling this substrate mixture over the catalyst, and thereby accumulating more 1:1 DAC/H_2_O product mixture for later separation (Figure 13). The latter promises to be relatively facile due to the absence of alcohol that forms an azeotrope with aqueous DAC.^80^

If this approach could be coupled to or even built into the reactor similar to the use of drying beds, much larger gains than for water removal alone are to be expected, as thermodynamically both DAC and H_2_O limit the equilibrium position of the system. This can be seen mathematically in eq 6 where both products form the numerator of the fraction. Figure 12B,D illustrates the gains in productivity achievable by this approach compared to water removal only (A and C), all relative to a single batch reaction as the first data point in each plot. By removing both products from the catalyst bed in the reactor, we calculate that up to 5 times greater MeOH conversion can be achieved over the same number of reaction/separation cycles than for water removal alone. Importantly, even with a mere 20% removal efficiency of both products, we calculate a 2-fold increase in MeOH conversion over water removal alone. The thermodynamic data for DEC show similar results: 20% removal of both products would accumulate close to 10% yield over 100 cycles (sufficient for distillative separation), whereas removal of water alone, even if 100% effective, would yield less than 5% yield (insufficient for distillative separation) over the same cycle number.

A variety of separation technologies may be evaluated to achieve this task, including CO_2_-induced phase splits,^72,73,81^ supercritical extraction,^82^ or pervaporation membranes. The latter have been used successfully for DMC/MeOH separations^83^ as well as two-step distillation–pervaporation separations in DEC synthesis from EtOH.^76^ Of course, all of these separation methods will have an associated energy cost for each cycle that will depend on the method chosen, concentration of the components, separation efficiency, and scale applied as previously investigated.^84−86^ Which of these technologies may prove viable for application will depend on a number of additional factors that are beyond the scope of this work (cost, lifetime, etc.), and a careful evaluation of compatibility with the reaction conditions and kinetics which are not considered in this thermodynamic analysis.

## Conclusions

Although dehydrating agents are widely used additives for the evaluation of catalyst materials in the formation of dialkyl carbonates from alcohol and CO_2_, they suffer from limitations such as complex hydrolysis kinetics and/or precipitation that make comparisons across different reports difficult. Most importantly, however, their use is nonsensical from thermodynamic, economic and environmental points of view, and as such, they do not contribute to an advancement of chemical CO_2_ utilization. A range of active catalysts have been developed for DAC formation, with commercial ceria remaining one of the most effective materials. Arguably, faster catalysts are not necessarily needed to advance this area, although catalysts with lower onset temperatures would allow for higher equilibrium yields that increase process productivity and facilitate product isolation. We have produced an accurate thermodynamic model that allows calculating equilibrium yields of DMC and DEC over a wide range of reaction conditions and predicting the effects of residual moisture as well as various product removal strategies. While water is widely considered the limiting byproduct in this reaction, removal of DAC product and water byproduct, which have been shown to be stable enough as a mixture on typical process timescales, is far more effective in accumulating practically useful DAC concentrations. We have demonstrated one of the most productive continuous flow systems for DMC and DEC formation from alcohol and CO_2_ to date and identified progressive surface M–O–M hydrolysis as the entry point into catalyst deactivation that is a major hurdle for process development. A new process model based on the continuous removal of both water and DAC product from the pressurized reactor effluent has been proposed, which along with sufficiently stable catalysts of perhaps also lower onset temperatures may make the direct carboxylation of alcohols an economically viable process as a meaningful contribution to practical CO_2_ utilization in the future.

## Supporting Information Available

Details of the experimental setup (incl. photographs), catalyst characterization (XRD, SEM, EDX data), analytical details and GC calibration, additional thermodynamic data, catalyst testing results and DMC hydrolysis experiments, and Arrhenius analysis of DMC formation (PDF)

## Notes

All data supporting this study is provided as Supporting Information accompanying this paper.

## Footnotes

a Note that these calculations do not give any indication of kinetics, i.e., the time required for each of these separation steps to proceed and the reactor content reequilibrate to reach these conversions.
