# Supplementary material for: Sustainable Synthesis of Dimethyl- and Diethyl Carbonate from CO2 in Batch and Continuous Flow—Lessons from Thermodynamics and the Importance of Catalyst Stability
Source: ACS Sustain Chem Eng. 2022 Apr 12;10(16):5243–57. doi: 10.1021/acssuschemeng.2c00291 (PMC9044503; doi:10.1021/acssuschemeng.2c00291)
Supplement: Supplementary file 1 — sc2c00291_si_001.pdf [file sc2c00291_si_001.pdf]

Supporting information

Sustainable Synthesis of Dimethyl- and Diethylcarbonate from CO_2 in Batch and Continuous Flow – Lessons from Thermodynamics and the Importance of Catalyst Stability

Matthew F. O'Neill,[a,b] Meenakshisundaram Sankar,[a]* Ulrich Hintermair[b]*

Number of pages: 22

Number of figures: 14

Number of tables: 4

[a] *Cardiff Catalysis Institute, School of chemistry, Cardiff University, Cardiff CF10 3AT, United Kingdom.*

[b] *Centre for Sustainable and Circular Technologies, University of Bath, Bath BA2 7AY, United Kingdom.*

* Corresponding Authors: Sankar@cardiff.ac.uk, U.Hintermair@bath.ac.uk

Table of contents

Figure S1: Photograph taken through view cell autoclave loaded with 2mL mixture of 2:1 methanol-DIC, pressurised to 70bar CO_2 at 40°C and heated to 120°C 4

Table S1: Catalysts trialled for batch reactions without a dehydrating agent........ 4

Figure S2: Normalised powder XRD of porous oxide catalysts synthesised by precipitation........ 5

Figure S3: Micrograph and EDX elemental mapping of a $Ce_{0.1}Zr_{0.9}O_2$ mixed metal oxide........ 5

Figure S4: SEM micrographs of mixed metal oxides synthesised in the course of this work........ 6

Table S2: Crystallite size, surface area and Lattice parameter for pure and mixed metal oxides synthesised by precipitation........ 8

Table S3: Enthalpies of formation (ΔH_f^0) and Standard entropy (S^0) in standard state for each component investigated in this work. 9

Figure S5: Calculated effect of wet starting material for the conversion of ethanol (left) and methanol (right) to DMC and DEC respectively 9

Figure S6: Schematic representation of the experimental setup used for continuous flow reactions in this work. 10

Figure S7: Relationship between catalyst contact time and DMC productivity at different CO_2 flow rates. 111

Table S4: Retention times for compounds detected by GC. 11

Figure S8: Example GC for DEC (top) and DMC (Bottom) with mesitylene as a standard........ 12

Figure S9: Calibration curve for DMC, plotted as DMC concentration against DMC peak area, normalised against the mesitylene peak area as internal standard........ 133

Figure S10: Calibration curve for DEC, plotted as DEC concentration against DEC peak area, normalised against the mesitylene peak area as internal standard........ 13

Figure S11: Cumulative DMC production over the course of the accelerated aging experiment in continuous flow 144

Figure S12: Hydrolysis of DMC at 80 and 100 °C as followed by ^{1}H qNMR spectroscopy........ 15

Figure S13: Catalytic formation and decomposition of dimethyl carbonate at 140, 120 and 100 °C. The dashed lines indicate the calculated equilibrium conversions at each temperature........ 166

Figure S14: 3-point Arrhenius plot of DMC formation using fitted k_{obs} for the formation of DMC from CO_2 and methanol over a commercial cerium oxide catalyst........ 17

Experimental 18

Powder X-ray diffraction... 18

Pure and mixed metal oxide preparation by precipitation..19

Surface area measurements..19

SEM-EDX...19

References 20

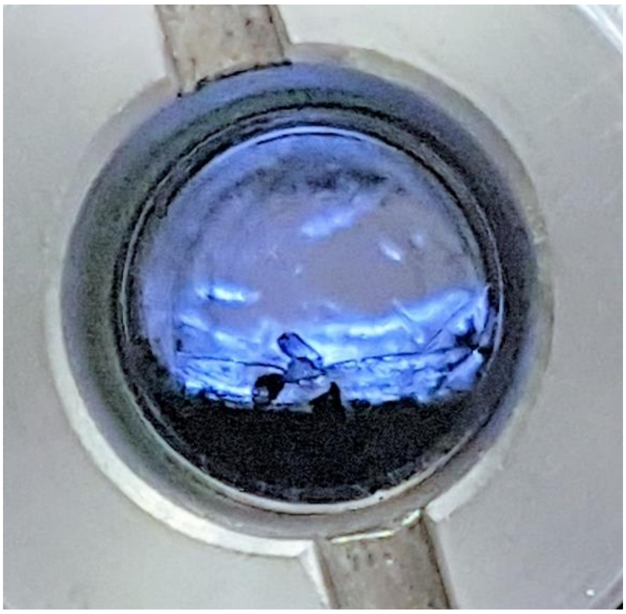

Figure S1: Photograph taken through view cell autoclave loaded with 2mL mixture of 2:1 methanol-DIC, pressurised to 70bar CO_2 at 40°C and heated to 120°C, showing the formation of diisopropyl urea crystals.

Table S1: Catalysts trailed for batch reactions without a dehydrating agent. Conditions: 24 h, 90 bar CO_2, 3 mL methanol, 0.03 g catalyst, 100 °C.

Entry	Catalyst	DMC Concentration (mM)	Conversion (%)
1	Commercial CeO_2	18.7	0.151
2	$Ce_{0.5}Zr_{0.5}O_2$	10.9	0.088
4	$Ce_{0.9}Al_{0.1}Ox$	7.56	0.061
5	CeO_2 (Precipitation)	7.01	0.057

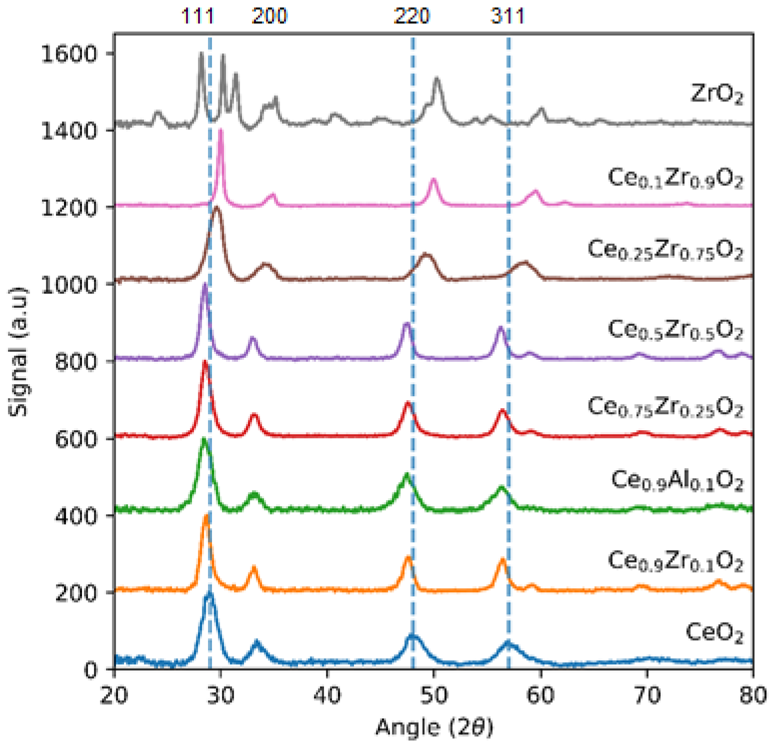

Figure S2: Normalised powder XRD of porous oxide catalysts synthesised by precipitation. Dashed lines show the shift of 2ϑ as the proportion of cerium decreases in the material.

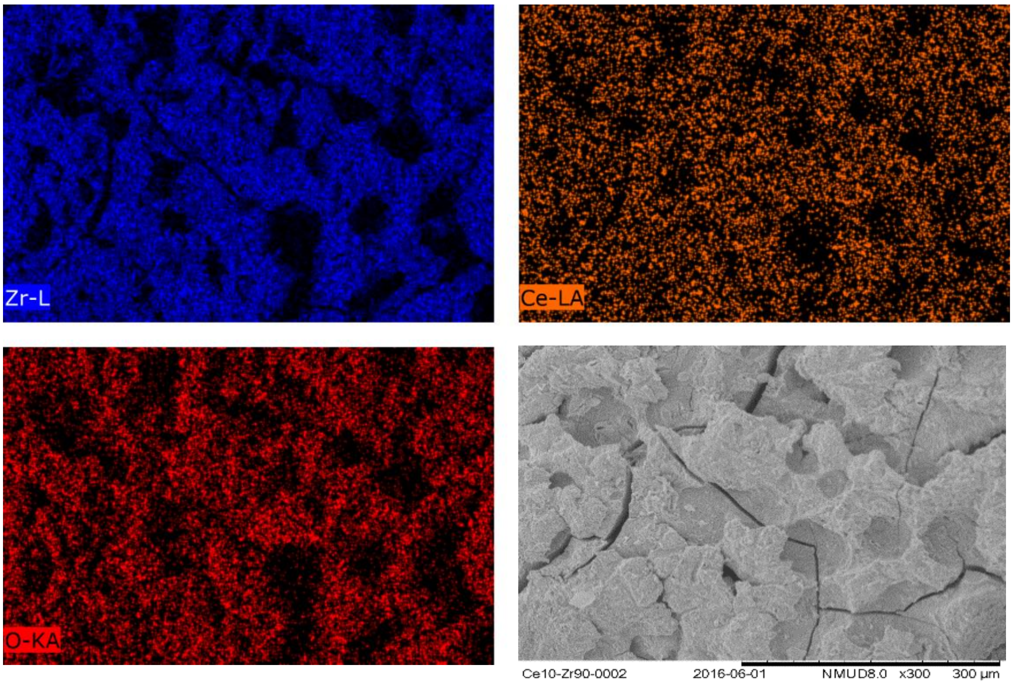

Figure S3: Micrograph and EDX elemental mapping of the same region of a $Ce_{0.1}Zr_{0.9}O_2$ mixed metal oxide showing intimate mixing of the ceria-zirconia solid solution.

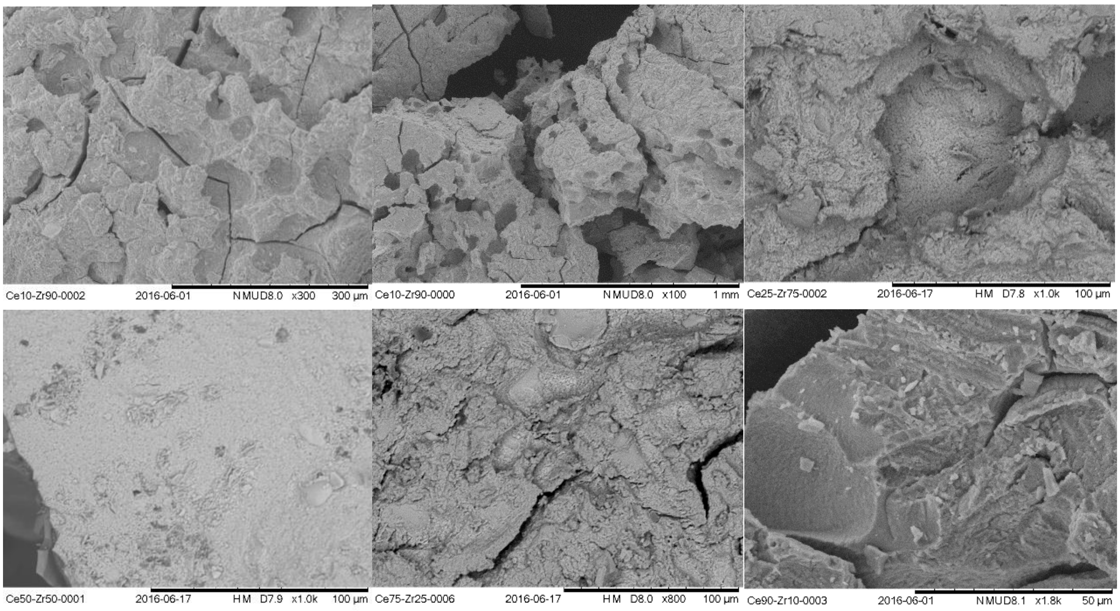

Figure S4: SEM micrographs of mixed metal oxides synthesised in the course of this work. Top row left to right: $Ce_{0.10}Zr_{0.90}O_2$, $Ce_{0.10}Zr_{0.90}O_2$, $Ce_{0.25}Zr_{0.75}O_2$. Bottom row left to right: $Ce_{0.5}Zr_{0.5}O_2$, $Ce_{0.75}Zr_{0.25}O_2$ and $Ce_{0.9}Zr_{0.1}O_2$.

Table S2: Crystallite size, surface area and Lattice parameter for pure and mixed metal oxides synthesised by precipitation. Crystallite size was calculated using the Scherrer equation, surface area using BET analysis and lattice parameter using the Braggs law for a cubic crystallite.

Catalyst	XRD crystallite size (nm)	Surface area ($m^2.g^{-1}$)	Lattice Parameter (Å)
ZrO_2	13.76	57	-
$Ce_{0.1}Zr_{0.9}O_2$	9.07	59	5.1485
$Ce_{0.25}Zr_{0.75}O_2$	5.20	88	5.2348
$Ce_{0.5}Zr_{0.5}O_2$	8.83	102	5.416
$Ce_{0.75}Zr_{0.25}O_2$	7.01	103	5.4082
$Ce_{0.9}Zr_{0.1}O_2$	7.87	108	5.3986
CeO_2	4.77	108	5.3625
$Ce_{0.9}Al_{0.1}O_y$	5.48	166	5.4136
Commercial	12.3	50	5.3977

Table S3: Enthalpies of formation (ΔH_f^0) and Standard entropy (S^0) in standard state for each component investigated in this work.

Component	ΔH_f^0 (kJ/mol)	Year published	Source	S^0 ($J \cdot K^{-1} \cdot mol^{-1}$)	Year published	Source
Dimethyl Carbonate $DMC_{(l)}$	-607.27	-	DIPPR project[1]	**218.7**	**-**	**DIPPR project[1]**
	-608.76	2015	NIST TDE 2015 (Knovel)[2]			
	**-613.78**	**2010**	**Lieno et al.[3]**			
	-614.53[a]	1991	NIST[4–7]			
	-595.73[a]	1991	NIST[4–6,8]			
	-607.60[b]	1972	NIST[4,9]			
Diethyl Carbonate $DEC_{(l)}$	-682.65	-	DIPPR project[1]	**293.3**	**-**	**DIPPR project[1]**
	**-681.58**	**2015**	**NIST TDE 2015 (Knovel)[10]**			
	-681.5	1972	NIST[4,11]			
	-724.17	1971	NIST[4,8]			
Methanol $MeOH_{(l)}$	-239.45	2015	NIST TDE 2015 (Knovel)[12]	**127.19**	**1971**	**NIST[13,14]**
	**-238.4**	**1972**	**NIST[4,15]**	126.8	1929	NIST[13,16]
	-239.5	1965	NIST[4,17]			
	-238.9	1960	NIST[4,18]			
Ethanol $EtOH_{(l)}$	-276.93	2015	NIST TDE 2015 (Knovel)[19]	**159.86**	**1977**	**NIST[13,20]**
	**-277**	**1965**	**NIST[4,17]**	161.21	1961	NIST[13,21]
	-277.6	1960	NIST[4,18]	160.7	1929	NIST[13,22]
Carbon dioxide $CO_{2(g)}$	**-393.51**	**2015**	**NIST TDE 2015 (Knovel)[23]**	**213.79**	**1998**	**NIST[13,24]**
	-393.52	1998	NIST[4,18]	213.785	1984	NIST[13,25]
Water $H_2O_{(l)}$	**-285.83**	**2015**	**NIST TDE 2015 (Knovel)[26]**	**69.95**	**1984**	**NIST[13,24]**
	-285.83	1998	NIST[4,27]			
	-285.83	1984	NIST[4,28]			

[a] Calculated from reaction enthalpy of the alcolysis of propylene carbonate. [b] calculated from the reaction enthalpy of the hydrolysis of tetramethyl orthocarbonate

Comment:

For the ΔH_f of DMC the value of -613.78 kJ/mol was used as this aligned most closely with experiment at the temperatures and pressures investigated. For the ΔH_f of DEC a mean was taken of all values reported excluding -724 kJ/mol which would imply a negative ΔG predicting a spontaneous reaction.

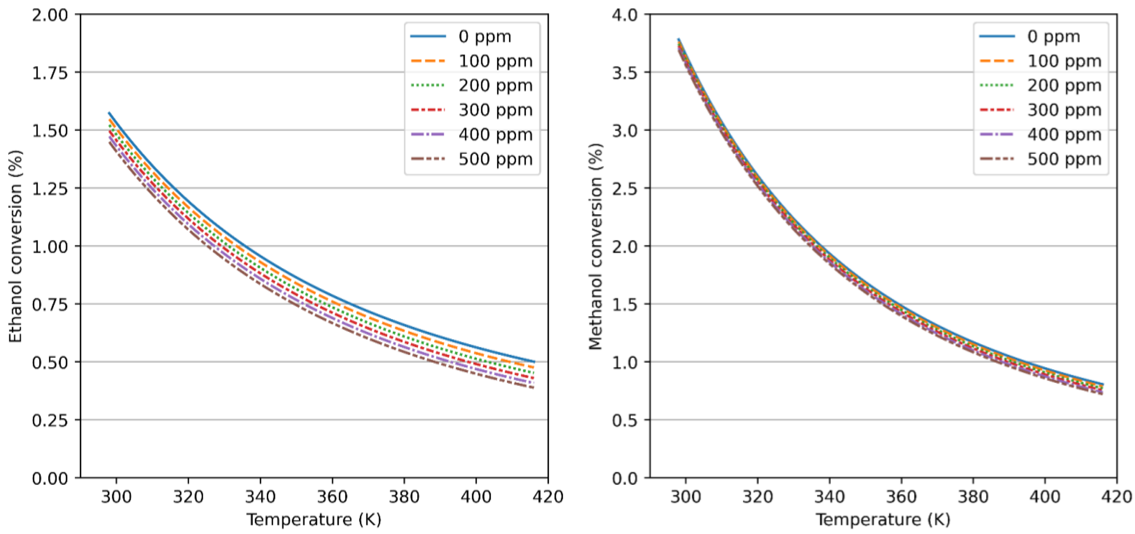

Figure S5: Calculated effect of wet starting material for the conversion of ethanol (left) and methanol (right) to DMC and DEC respectively. Conditions: 0.66g/mL CO_2 (110 bar at 313 K).

Comment:

Using the model developed for this work with a starting concentration of products, the effect of wet starting material could be determined. Water ppm was calculated on a mass/mass where 500ppm is 500 millionths of a gram of water per gram of alcohol. This effect is not large for relatively dry methanol (100–200ppm) but a larger effect is predicted for ethanol, conversion dropping from 0.5% (0 ppm) to 0.3% (500 ppm) at 413K. The model is available at https://github.com/DeemoONeill/Thermodynamics_class under an MIT licence.

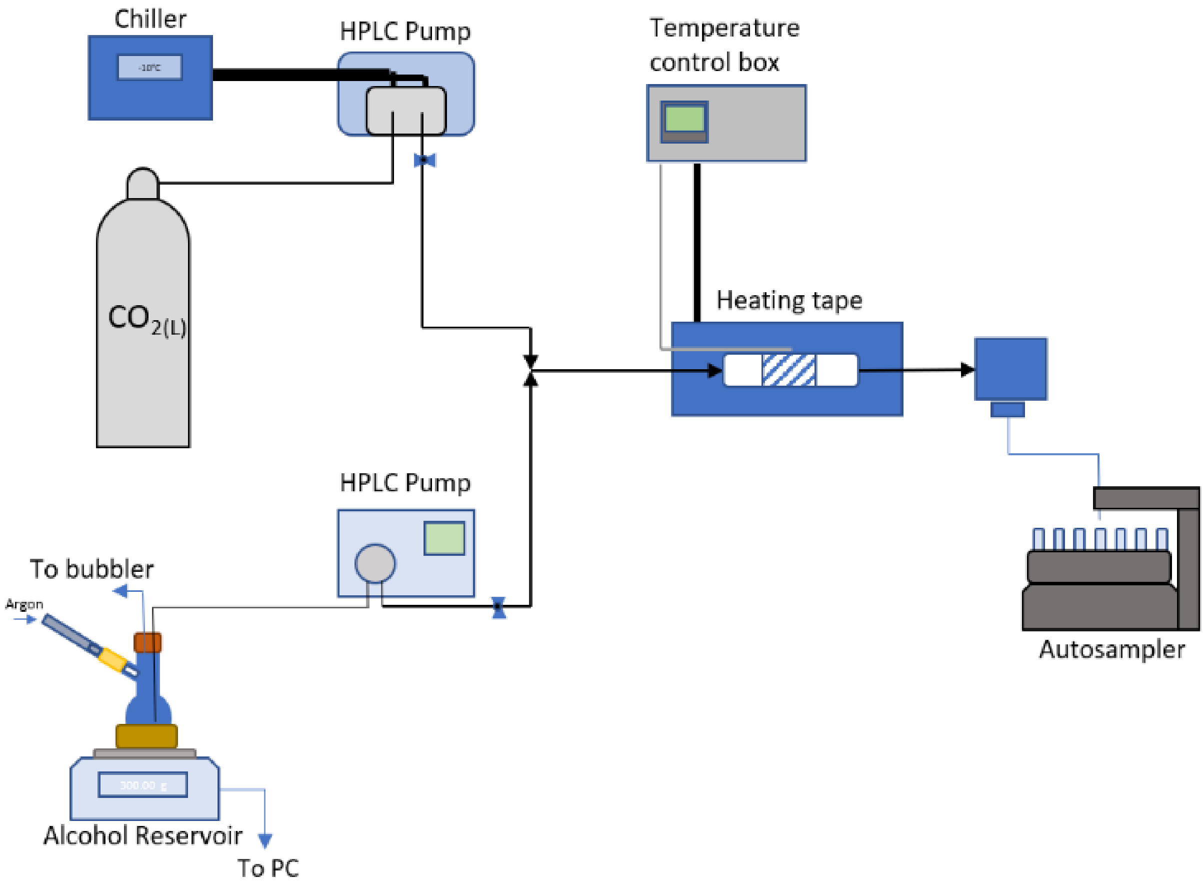

Figure S6: Schematic representation of the experimental setup used for continuous flow reactions in this work.

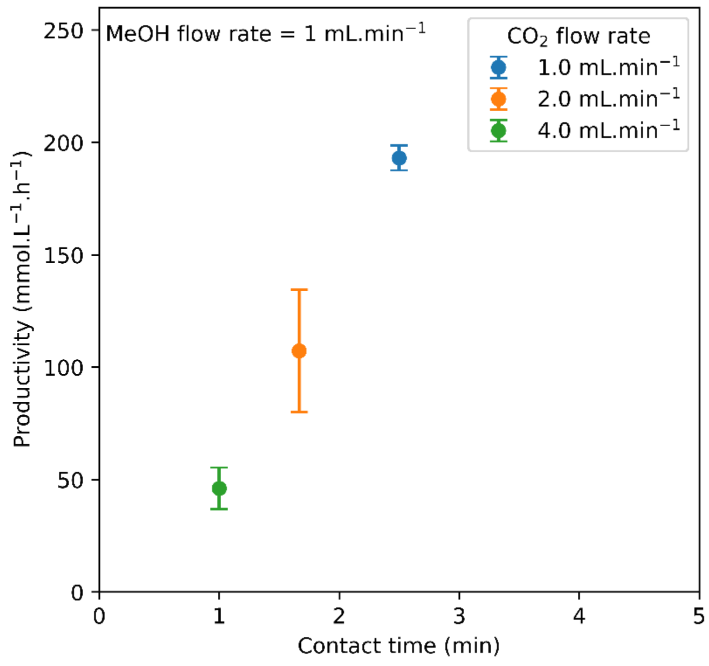

Figure S7: Relationship between catalyst contact time and DMC productivity at different CO_2 flow rates. Reaction conditions: 3g CeO_2, 140°C, 200 bar CO_2, 1 mL.min^{-1} MeOH.

Table S4: Retention times for compounds detected by GC.

Compound	Retention time (min)	GC	Column
Methanol	6.43	Varian 3900	CP-Sil 5CB
Dimethyl carbonate	10.6	Varian 3900	CP-Sil 5CB
1,3,5-trimethoxybenzene	44.8	Varian 3900	CP-Sil 5CB
Methanol	3.61	Shimadzu GC-2010 plus	BP20
Ethanol	4.02	Shimadzu GC-2010 plus	BP20
Dimethyl carbonate	5.01	Shimadzu GC-2010 plus	BP20
Diethyl carbonate	8.06	Shimadzu GC-2010 plus	BP20
Mesitylene	12.4	Shimadzu GC-2010 plus	BP20

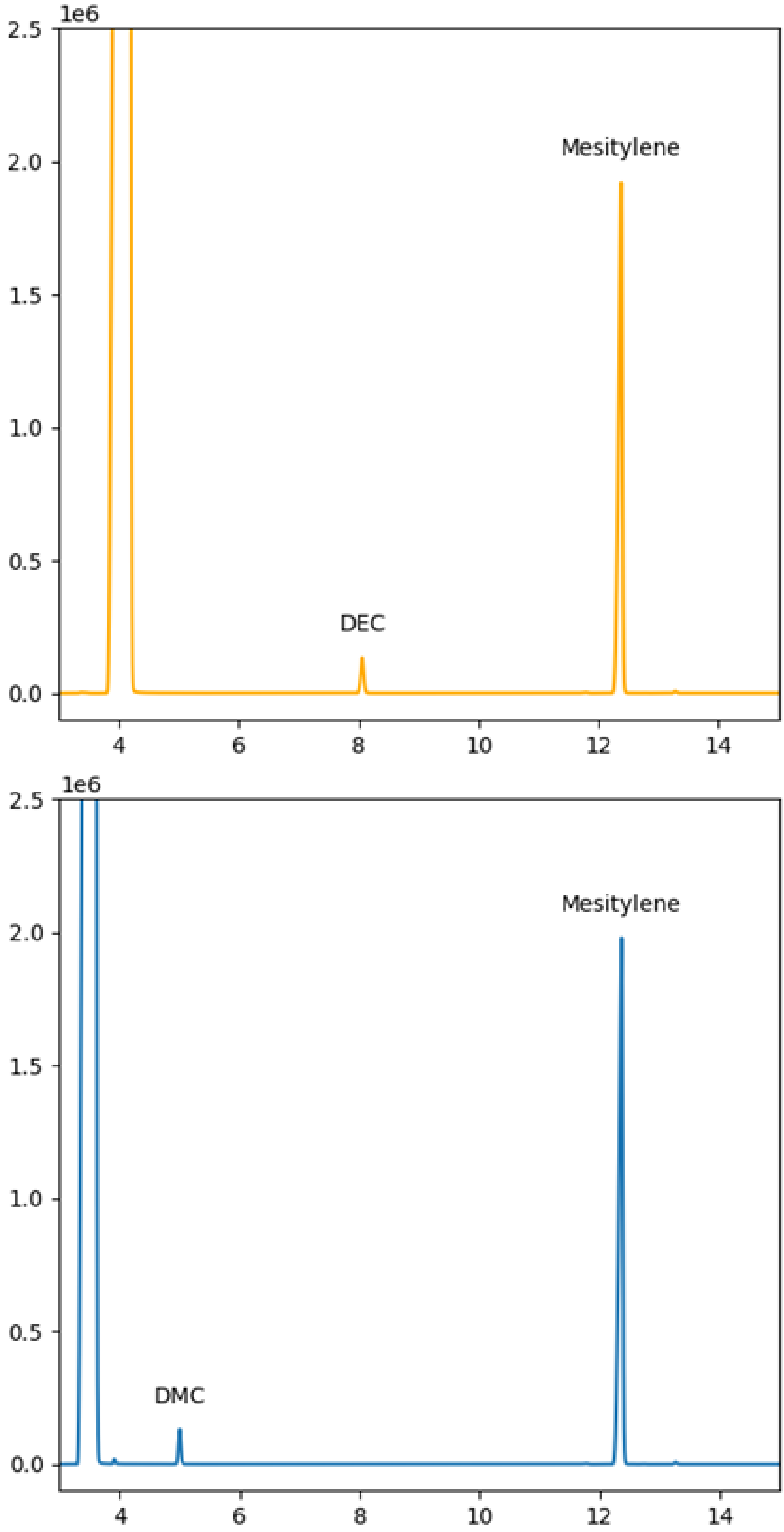

Figure S8: Example GC for DEC (top) and DMC (bottom) with mesitylene as a standard.

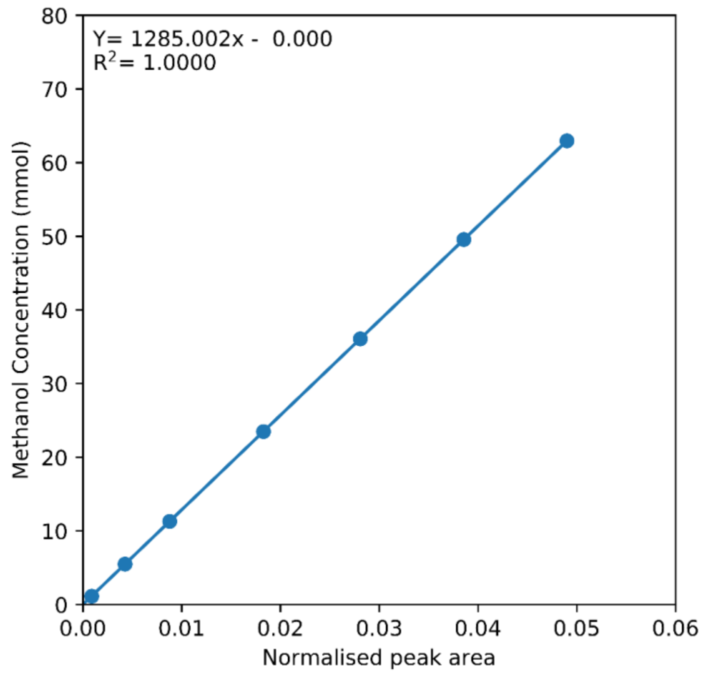

Figure S9: Calibration curve for DMC, plotted as DMC concentration against DMC peak area, normalised against the mesitylene peak area as internal standard.

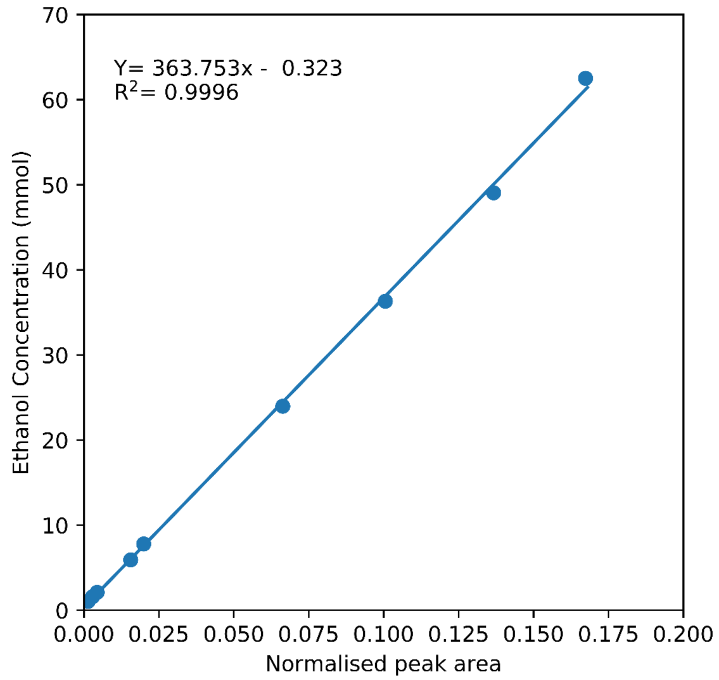

Figure S10: Calibration curve for DEC, plotted as DEC concentration against DEC peak area, normalised against the mesitylene peak area as internal standard.

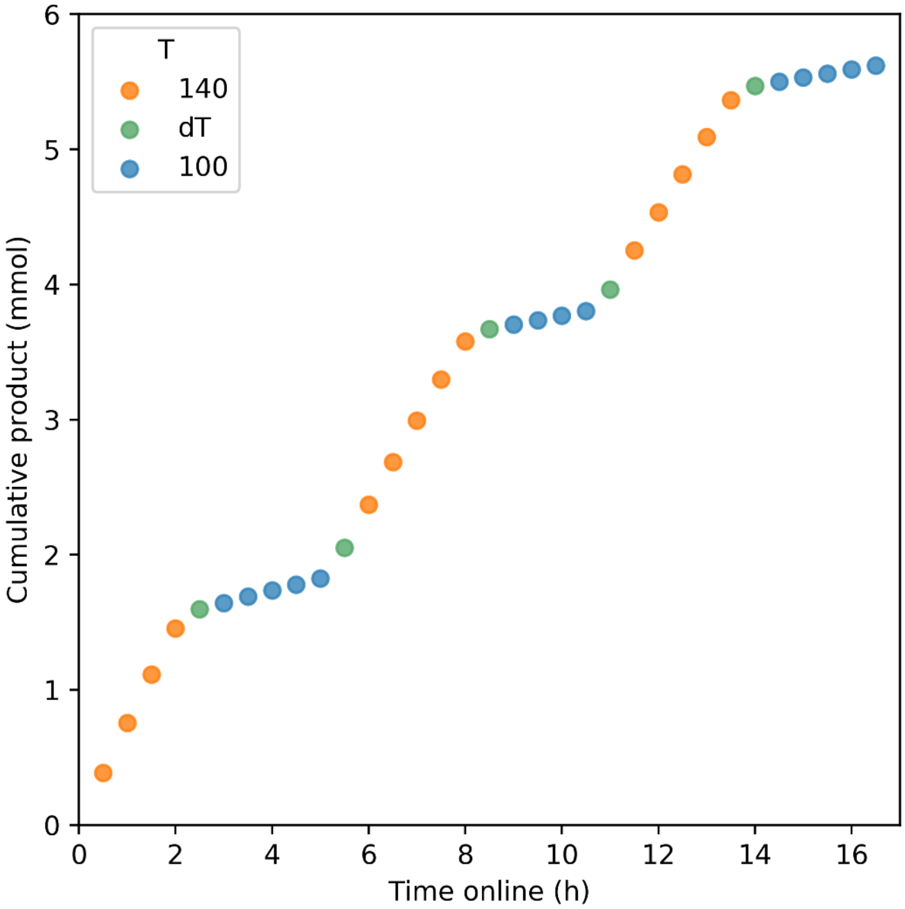

Figure S11: Cumulative DMC production over the course of the accelerated aging experiment in continuous flow. Conditions: 100 and 140 °C, 1 ml.min^{-1} CO_2 at 200 bar, 0.2 ml.min^{-1} MeOH.

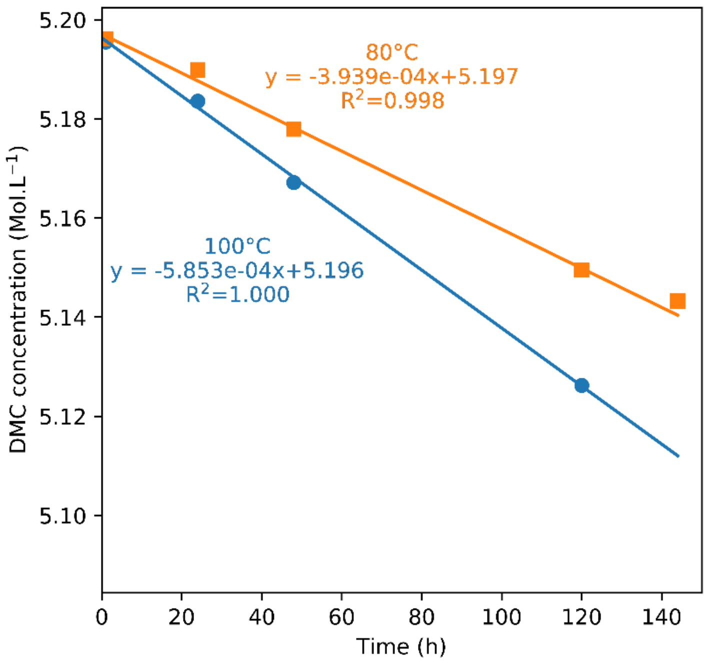

Figure S12: Hydrolysis of DMC at 80 and 100 °C as followed by ^{1}H qNMR spectroscopy. Conditions: 12 mmol DMC, 13 mmol H_2O (adjusted to pH 3 with HNO_3), 1 mL DMSO, 0.05 mL d_6-DMSO.

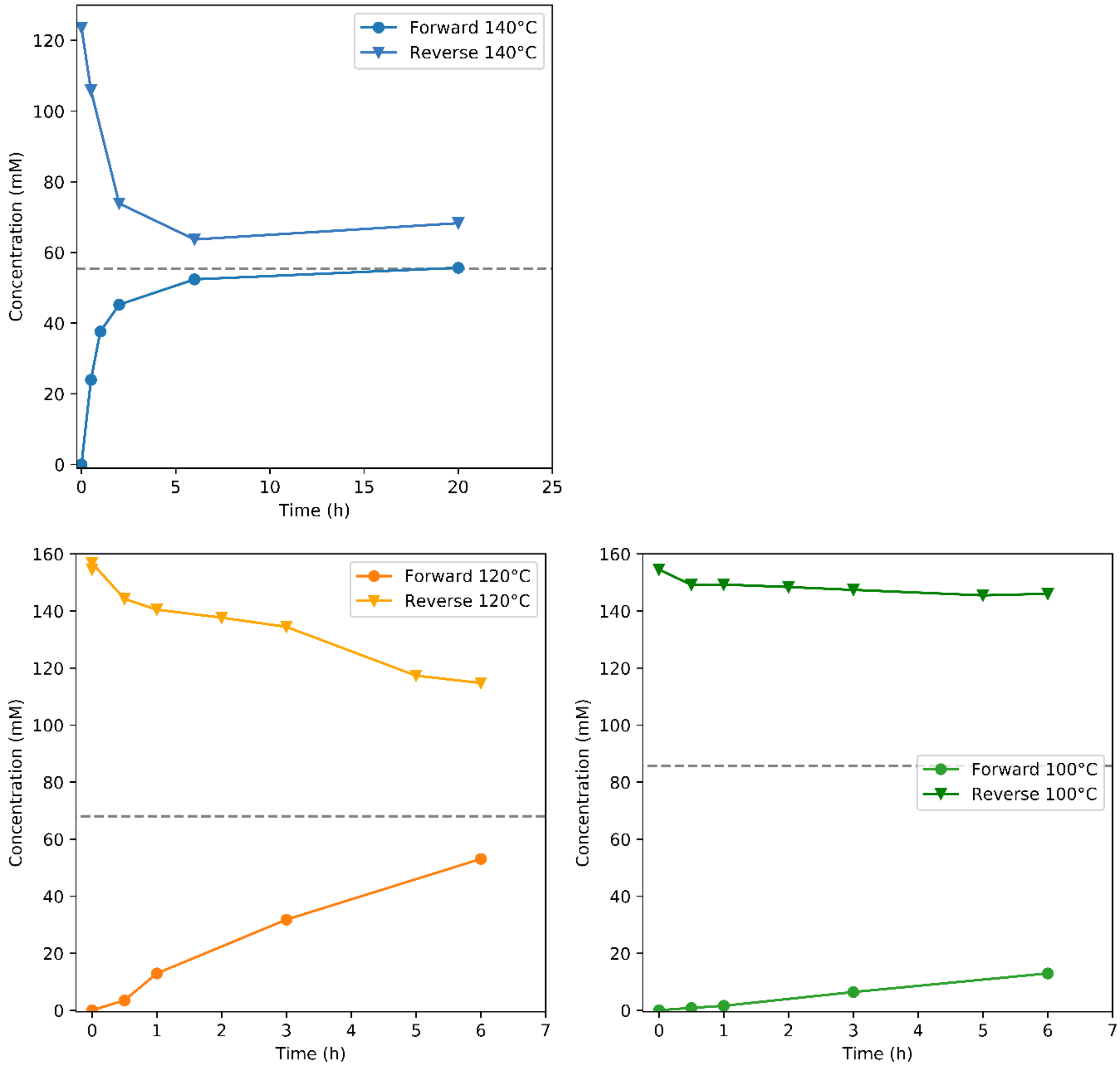

Figure S13: Catalytic formation and decomposition of dimethyl carbonate at 140, 120 and 100 °C. The dashed lines indicate the calculated equilibrium conversions at each temperature. Reaction conditions, forward: 0.3g CeO_2, 70 bar CO_2 at 40 °C, 5 mL dry methanol. Reverse: 0.3g CeO_2, 5 mL stock solution (124 or 158mM DMC and water in methanol), 70 bar CO_2 at 40 °C. Lines added as a guide to the eye.

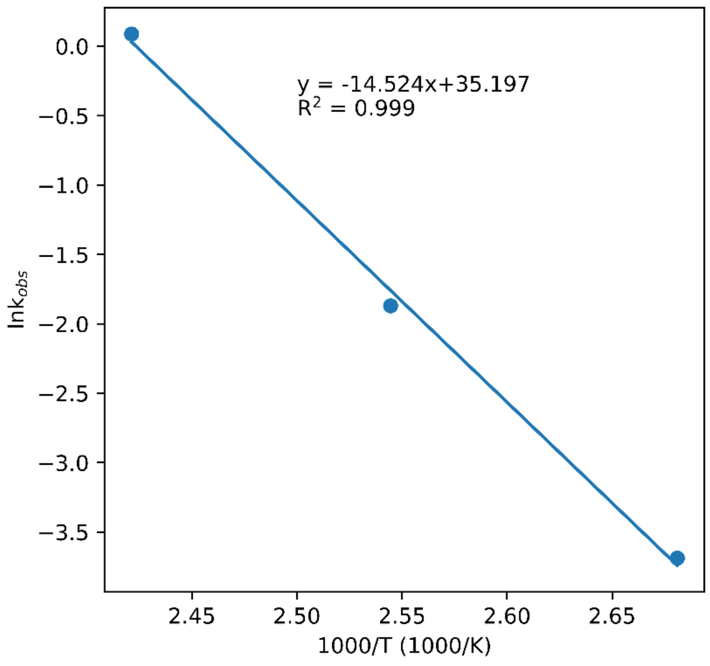

Figure S14: 3-point Arrhenius plot of DMC formation using fitted k_{obs} for the formation of DMC from CO_2 and methanol over a commercial cerium oxide catalyst.

Comment: The three point Arrhenius plot gives a E_a of 120 kJ/mol which is within 2.5% of the value reported by Santos *et al.*[29] However due to the fitting of the experimental data this carries an error of ~10%. The equation that was fitted to the data is the reversible first order equation Eq. 1.[30]

Eq. 1 $$[DMC] = ([DMC]_0 - [DMC]_{eq}) \cdot e^{-k_{obs} \cdot t} + [DMC]_{eq}$$

Where:

$[DMC]_0$ = Concentration of DMC at t = 0 (mM)

$[DMC]_{eq}$ = equilibrium concentration of DMC (mM)

K_{obs} = observed rate constant (mM/h)

t = time (h)

This allows for fitting of both forward and reverse reactions, though should only be used within a small range of concentrations above and below the equilibrium as there will be a maximum achievable rate which will not be accounted for in the above equation.

Experimental

Powder X-ray diffraction

Samples made by precipitation were loaded into a sample holder and analysed using STOI STADI P with a Cu K-α source and scanned from 10-80 2θ.

Crystallite size was calculated using the Scherrer equation:

Eq. 2 $$\tau = \frac{K\lambda}{\beta \cos\theta}$$

Full width at half maximum (FWHM) β was determined in python using the SciPy package[31]

d-spacing was calculated using Braggs law:

Eq. 3 $$\lambda = 2d_{hkl} sin\theta_{hkl}$$

For the cubic crystallites the d values were calculated by:

Eq. 4 $$\frac{1}{d^2} = \frac{h^2+k^2+l^2}{a^2}$$

Which can be combined to give lattice parameter a:

Eq. 5 $$a^2 = \frac{\lambda^2}{4sin^2\theta}(h^2 + k^2 + l^2)$$

Where:

τ = crystallite size (nm)

K = dimensionless shape factor

β = Full width at half maximum (radians)

θ = Bragg angle (radians)

λ = x-ray wavelength (Å)

h, k, l = miller indices of the Bragg plane

a = lattice parameter (Å)

d = interplanar spacing (Å)

Pure and mixed metal oxide preparation by precipitation

$Ce(NO_3)_3 \cdot 6H_2O$ (Sigma), $ZrO(NO_3)_2 \cdot XH_2O$ (Sigma) or a mixture of the two were loaded into a 50ml round bottom flask along with 2.99 g Pluronic® P104 (BASF) with 40ml ethanol or deionised water and stirred for 3h at room temperature. After 3 hours 14 mL of 35 % ammonia was added dropwise over 30 minutes under constant stirring until pH 11 was achieved. This was then aged under constant stirring for 18 h before being filtered on a Buchner funnel and washed with deionised water. The sample was then dried under vacuum for 3 h, the resultant powder was Calcined at 450 °C for 4 h at 1 °C/min ramp rate.

Surface area measurements

Surface area was measured by nitrogen sorption isotherms at -196°C on a on Quantachrome NOVA 2200e. Surface area was calculated using the Brunauer−Emmett−Teller (BET) method. Samples were pre-treated for 1 h at 300°C under vacuum.

SEM-EDX

SEM Micrographs were taken using a Hitachi TM3030Plus tabletop microscope equipped with a Bruker EDX detector. Samples were analysed using back scatter electrons at 15 kV. Surface composition was determined using EDX composition of ceria/zirconia samples.

References

(1) Design Institute for Physical Properties. DIPPR Project 801 - Full Version. Design Institute for Physical Property Research/AIChE https://app.knovel.com/hotlink/toc/id:kpDIPPRPF7/dippr-project-801-full/dippr-project-801-full.

(2) Knovel; National Institute of Standards and Technology. KDA - dimethyl carbonate https://app.knovel.com/web/poc/ms/profile.html?cid=kcE6MU25U9&prop=prEFO (accessed Jul 2, 2020).

(3) Leino, E.; Mäki-Arvela, P.; Eta, V.; Murzin, D. Y.; Salmi, T.; Mikkola, J. P. Conventional Synthesis Methods of Short-Chain Dialkylcarbonates and Novel Production Technology via Direct Route from Alcohol and Waste CO2. *Appl. Catal. A Gen.* **2010**, *383* (1–2), 1–13. https://doi.org/10.1016/j.apcata.2010.05.046.

(4) Afeefy, H. Y.; Liebman, J. F.; Stein, S. E. Neutral Thermochemical Data. In *NIST Chemistry WebBook, NIST Standard Reference Database Number 69*; Linstrom, P. J., Mallard, W. G., Eds.; National Institute of Standards and Technology: Gaithersburg MD, 20899. https://doi.org/10.18434/T4D303.

(5) Knauth, P.; Sabbah, R. Energetics of Inter. and Intramolecular Bonds in Alkanediols. Iv. the Thermochemical Study of 1,2-Alkanediols at 298.15 K. *Thermochim. Acta* **1990**, *164* (C), 145–152. https://doi.org/10.1016/0040-6031(90)80431-W.

(6) Zhang, S.; Luo, Y. Studies on Kinetics and Technological Conditions of Synthesis of Dimethyl Carbonate. *huaxue fanying gongcheng yu gongyi* **1991**, *7* (1), 10–19.

(7) Vasil'eva, T. F.; Zhil'tosova, E. N.; Vvedenski, A. A. Enthalpies of Combustion of Alkylene Carbonates. *Russ. J. Phys. Chem. (Engl. Transl.)* **1972**, *46*, 316.

(8) Joncich, M. J.; Choi, J. K. Heats of Combustion, Heats of Formation and Vapor Pressures of Some Organic Carbonates Estimation of Carbonate Group Contribution to Heat of Formation. *J. Chem. Eng. Data* **1971**, *16* (1), 87–90. https://doi.org/10.1021/je60048a026.

(9) Wiberg, K. B. *Energies of Organic Compounds*; 1989.

(10) Knovel; National Institute of Standards and Technology. KDA - diethyl carbonate https://app.knovel.com/web/poc/ms/profile.html?cid=kcNK0LV7BQ (accessed Jul 2, 2020).

(11) Månsson, M. Enthalpies of Combustion and Formation of Ethyl Propionate and Diethyl Carbonate. *J. Chem. Thermodyn.* **1972**, *4* (6), 865–871. https://doi.org/10.1016/0021-9614(72)90008-0.

(12) Knovel; National Institute of Standards and Technology. KDA - methanol https://app.knovel.com/web/poc/ms/profile.html?cid=kcY4PHE01W (accessed Jul 2, 2020).

(13) Domalski, E. S.; Hearing, E. D. Condensed Phase Heat Capacity Data. In *NIST Chemistry WebBook, NIST Standard Reference Database Number 69*; Linstrom, P. J., Mallard, W. G., Eds.; National Institute of Standards and Technology: Gaithersburg MD, 20899. https://doi.org/10.18434/T4D303.

(14) Carlson, H. G.; Westrum, E. F. Methanol: Heat Capacity, Enthalpies of Transition and Melting, and Thermodynamic Properties from 5-300°K. *J. Chem. Phys.* **1971**, *54* (4), 1464–1471. https://doi.org/10.1063/1.1675039.

(15) Baroody, E E; Carpenter, G. A. Heat of Formation of Propellant Compounds (U). *Rpt. Nav. Ordanance Syst. Command Task No. 331-003/067-1/UR2402-001* **1972**, 1–9.

(16) Kelley, K. K. The Heat Capacity of Methyl Alcohol from 16K to 298K and the Corresponding Entropy and Free Energy. *J. Am. Chem. Soc.* **1929**, *51* (9), 180–187. https://doi.org/10.1021/ja01384a018.

(17) Chao, J.; Rossini, F. D. Heats of Combustion, Formation, and Isomerization of Nineteen Alkanols. *J. Chem. Eng. Data* **1965**, *10* (4), 374–379. https://doi.org/10.1021/je60027a022.

(18) Green, J. H. S. Revision of the Values of the Heats of Formation of Normal Alcohols. *Chem. Ind.* **1960**, 1215–1216.

(19) Knovel; National Institute of Standards and Technology. KDA - ethanol https://app.knovel.com/web/poc/ms/profile.html?cid=kcQ0NB21PG (accessed Jul 2, 2020).

(20) Haida, O.; Suga, H.; Seki, S. Calorimetric Study of the Glassy State XII. Plural Glass-Transition Phenomena of Ethanol. *J. Chem. Thermodyn.* **1977**, *9* (12), 1133–1148. https://doi.org/10.1016/0021-9614(77)90115-X.

(21) Green, J. H. S. Thermodynamic Properties of Organic Oxygen Compounds. *Q. Rev. Chem. Soc.* **1961**, *15* (2), 125–152. https://doi.org/10.1039/qr9611500125.

(22) Kelley, K. K. The Heat Capacities of Ethyl and Hexyl Alcohols from 16°K to 298°K and the Corresponding Entropies and Free Energies. *J. Am. Chem. Soc.* **1929**, *51* (4), 779–786. https://doi.org/10.1021/ja01379a022.

(23) Knovel; National Institute of Standards and Technology. KDA - carbon dioxide https://app.knovel.com/web/poc/ms/profile.html?cid=kcN22FE3G5 (accessed Jul 2, 2020).

(24) Cox, J. D.; Wagman, D. D.; Medvedev, V. A. CODATA Key Values for Thermodynamics. **1984**, 1.

(25) Chase, M. W. . J. NIST-JANAF Themochemical Tables, Fourth Edition. *J. Phys. Chem. Ref. Data, Monogr. 9* **1998**, 1–1951.

(26) Knovel; National Institute of Standards and Technology. KDA - water https://app.knovel.com/web/poc/ms/profile.v?cid=kcC4EC6H01&prop=prEFO (accessed Jul 2, 2020).

(27) Cox, J. D.; Wagman, D. D.; Medvedev, V. A. CODATA Key Values for Thermodynamics. *Netsu Sokutei* **1976**, *3* (2), 61–62. https://doi.org/10.11311/jscta1974.3.61.

(28) Chase, M.W., J. NIST-JANAF Themochemical Tables, Fourth Edition. *J. Phys. Chem. Ref. Data, Monogr. 9* **1998**, 1–1951.

(29) Santos, B. A. V.; Pereira, C. S. M.; Silva, V. M. T. M.; Loureiro, J. M.; Rodrigues, A. E. Kinetic Study for the Direct Synthesis of Dimethyl Carbonate from Methanol and CO2 over CeO2 at High Pressure Conditions. *Appl. Catal. A Gen.* **2013**, *455*, 219–226. https://doi.org/10.1016/j.apcata.2013.02.003.

(30) Nnaji, N. J.; Ani, J. U.; Ekwonu, A. M. The Solution of Reversible First Order Reaction Equation Revisited. *Acta Chim. Pharm. Indica* **2013**, *3* (3), 212–218.

(31) Virtanen, P.; Gommers, R.; Oliphant, T. E.; Haberland, M.; Reddy, T.; Cournapeau, D.; Burovski, E.; Peterson, P.; Weckesser, W.; Bright, J.; van der Walt, S. J.; Brett, M.; Wilson, J.; Millman, K. J.; Mayorov, N.; Nelson, A. R. J.; Jones, E.; Kern, R.; Larson, E.; Carey, C. J.; Polat, İ.; Feng, Y.; Moore, E. W.; VanderPlas, J.; Laxalde, D.; Perktold, J.; Cimrman, R.; Henriksen, I.; Quintero, E. A.; Harris, C. R.; Archibald, A. M.; Ribeiro, A. H.; Pedregosa, F.; van Mulbregt, P.; Vijaykumar, A.; Bardelli, A. Pietro; Rothberg, A.; Hilboll, A.; Kloeckner, A.; Scopatz, A.; Lee, A.; Rokem, A.; Woods, C. N.; Fulton, C.; Masson, C.; Häggström, C.; Fitzgerald, C.; Nicholson, D. A.; Hagen, D. R.; Pasechnik, D. V; Olivetti, E.; Martin, E.; Wieser, E.; Silva, F.; Lenders, F.;

Wilhelm, F.; Young, G.; Price, G. A.; Ingold, G.-L.; Allen, G. E.; Lee, G. R.; Audren, H.; Probst, I.; Dietrich, J. P.; Silterra, J.; Webber, J. T.; Slavič, J.; Nothman, J.; Buchner, J.; Kulick, J.; Schönberger, J. L.; de Miranda Cardoso, J. V.; Reimer, J.; Harrington, J.; Rodríguez, J. L. C.; Nunez-Iglesias, J.; Kuczynski, J.; Tritz, K.; Thoma, M.; Newville, M.; Kümmerer, M.; Bolingbroke, M.; Tartre, M.; Pak, M.; Smith, N. J.; Nowaczyk, N.; Shebanov, N.; Pavlyk, O.; Brodtkorb, P. A.; Lee, P.; McGibbon, R. T.; Feldbauer, R.; Lewis, S.; Tygier, S.; Sievert, S.; Vigna, S.; Peterson, S.; More, S.; Pudlik, T.; Oshima, T.; Pingel, T. J.; Robitaille, T. P.; Spura, T.; Jones, T. R.; Cera, T.; Leslie, T.; Zito, T.; Krauss, T.; Upadhyay, U.; Halchenko, Y. O.; Vázquez-Baeza, Y.; SciPy 1.0 Contributors. SciPy 1.0: Fundamental Algorithms for Scientific Computing in Python. *Nat. Methods* **2020**. https://doi.org/10.1038/s41592-019-0686-2.
